# Tropical Indian Ocean drives Hadley circulation change in a warming climate

**DOI:** 10.1093/nsr/nwae375

**Published:** 2024-10-22

**Authors:** Yong Sun, Gilles Ramstein, Alexey V Fedorov, Lin Ding, Bo Liu

**Affiliations:** State Key Laboratory of Tibetan Plateau Earth System, Environment and Resources (TPESER), Institute of Tibetan Plateau Research, Chinese Academy of Sciences, Beijing 100101, China; Laboratoire des Sciences du Climat et de l’Environnement, LSCE/IPSL, CEA‐CNRS‐UVSQ, Université Paris‐Saclay, Gif-sur-Yvette 91191, France; Laboratoire des Sciences du Climat et de l’Environnement, LSCE/IPSL, CEA‐CNRS‐UVSQ, Université Paris‐Saclay, Gif-sur-Yvette 91191, France; Department of Earth and Planetary Sciences, Yale University, New Haven, CT 06511, USA; LOCEAN/IPSL, Sorbonne University, Paris 75005, France; State Key Laboratory of Tibetan Plateau Earth System, Environment and Resources (TPESER), Institute of Tibetan Plateau Research, Chinese Academy of Sciences, Beijing 100101, China; Plateau Atmosphere and Environment Key Laboratory of Sichuan Province, School of Atmospheric Sciences, Chengdu University of Information Technology, Chengdu 610225, China

**Keywords:** Hadley circulation, Paris Agreement, tropical Indian Ocean, tropical Pacific Ocean, Robust responses and sources of uncertainty

## Abstract

The weakening and poleward expansion of the Hadley circulation (HC) are considered robust responses of atmospheric meridional circulation to anthropogenic warming. Climate impacts arising from these changes enhance drought conditions and reduce food production in the affected regions. Therefore, understanding the mechanisms of HC changes is critical to anticipating the resultant climate risks. First, we demonstrate that robust future HC changes in boreal winter, and the uncertainty in their future projections, are both largely related to sea surface temperature (SST) warming. Next, we investigate the impact of anthropogenic regional ocean warming on the future HC. Accordingly, we conduct a large ensemble of individual ocean basin perturbation experiments at 1.5°C, 2°C, and 3°C warming thresholds (as in the Paris Agreement). These experiments highlight (i) the leading role of tropical Indian Ocean warming in HC changes and (ii) inter-model differences in tropical Pacific warming as a source of uncertainty in HC projections.

## Teaser

This study examines how future warming in different ocean basins may affect the Hadley circulation a crucial component of the global climate system. It is found that warming in the tropical Indian Ocean is the primary driver of future changes in this circulation, while variations in tropical Pacific warming contribute to uncertainties in climate projections. These findings enhance our understanding of the potential impacts of global warming on atmospheric circulation.

## INTRODUCTION

The Hadley circulation (hereafter HC), a critical component of atmospheric meridional overturning circulation, is characterized by rising air near the equator and sinking air around 30$^\circ $N and 30$^\circ $S [1]. It shapes the tropical planetary-scale rain belt (i.e. the Intertropical Convergence Zone, ITCZ) and the subtropical arid zones [2], transporting energy poleward, but moisture equatorward. Widening and weakening of HC are robust atmospheric responses to global warming [3–6]. In particular, exacerbated subtropical aridity and the risk of subtropical drought invading mid-latitudes, both effects due to HC expansion [7–13], are of utmost socio-ecological concern to the public [14–16]. It is therefore essential to unravel the mechanisms underpinning future changes in HC in order to better predict climate impacts from changes in the HC.

As the observed warming of the ocean breaks records more and more frequently [17], this study aims to assess the potential climate impacts of future sea surface temperature (SST) warming due to increases in anthropogenic greenhouses gas (GHG) emissions. Future projections of SST changes and associated climate influences are typically evaluated using a broad range of climate model experiments, under different atmospheric CO_2_ rise scenarios. GHGs affect climate both directly and indirectly, specifically via the direct effect of infrared radiation and indirect effect of SST changes. Decomposition of GHG forced influences into direct and indirect effects using Atmospheric General Circulation Models (AGCMs) has been used previously to understand the tropical atmospheric meridional overturning circulation responses to increasing CO_2_ in future projections [18,19]. However, no consensus has been achieved regarding the relative importance of direct and indirect effects in shaping the future HC changes [18–27]. Therefore, the relative roles of direct radiative forcing and SST changes in future HC changes need to be reexamined.

In addition, specific contributions of individual ocean basin warmings to future HC changes remain unclear as well. There is also considerable inter-model spread in the response of tropical atmospheric circulation, especially HC, to future ocean warming patterns [28,29], and which ocean basin warming contributes to such uncertainty needs to be clarified.

Accordingly, in this study, we aim to (1) quantify the relative importance of direct and indirect effects in shaping the future HC changes in boreal winter while focusing on the HC edges in the Northern and Southern Hemispheres (Lat_NHC_ and Lat_SHC_), the position of the ascending branch of HC (corresponding to ITCZ, a narrow belt of intense rainfall near the equator, hereafter Lat_ITCZ_) and the intensity of HC in the Northern Hemisphere (Intensity_NHC_); (2) isolate the roles of individual ocean basins and the key mechanisms that control Lat_ITCZ_, Lat_NHC_, Lat_SHC_ and Intensity_NHC_ under the temperature thresholds of 1.5°C, 2°C and 3°C above the pre-industrial level; (3) investigate the source of inter-model spread in future HC projections associated with SST warming and uncover the underlying cause of this uncertainty.

## RESULTS

### Contribution of direct radiative forcing versus SST changes to future HC changes

**Figure 1.**
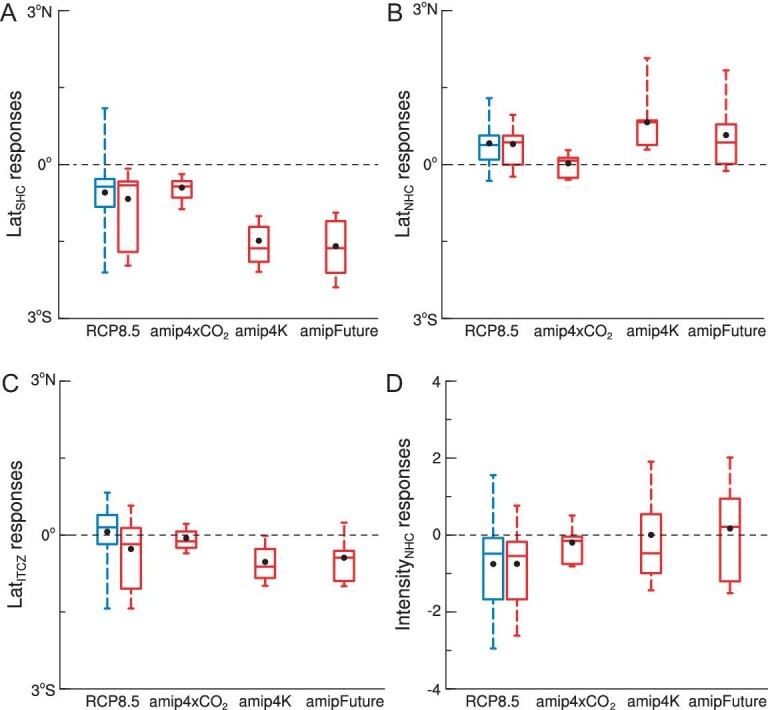
The impacts of CO_2_ radiative forcing versus ocean warming on the Hadley circulation and the ITCZ. We focus on changes in (A) Southern and (B) Northern Hadley cell extent (Lat_SHC_ and Lat_NHC_), (C) ITCZ position (Lat_ITCZ_), and (D) Northern Hadley cell intensity (Intensity_NHC_; units, 10^10^ kg$\cdot $s^−1^) during boreal winter (December-January-February, DJF). We compare the full effect of anthropogenic greenhouse gases under RCP8.5 scenario (RCP8.5 minus historical simulations) with the direct radiative effect (amip4xCO_2_ minus amip), the indirect effect due to SST changes with uniform global 4 K warming (amip4K minus amip), and the effect of non-uniform pattern of future global warming (amipFuture minus amip). Black dots are the results of multi-model ensemble (MME) mean. Box-whisker plots show the minimum, 25th, 50th, 75th percentiles and maximum values. Blue box-whisker plots are derived from all the models available for RCP8.5 projection, with 11 models available for RCP8.5 projection (red box-whisker) that can be used for comparison with the direct radiative effect, effects of uniform 4 K warming and SST warming patterns (red box-whisker). The results show that the displacement of Hadley cell and ITCZ, as well as the uncertainty range in the strength of Hadley cell in the Northern Hemisphere, are largely controlled by SST changes.

To assess the relative contributions of GHG radiative forcing and indirect effects due to SST warming to future changes of HC under the RCP8.5 scenario, we use six types of CMIP5 experiments. These experiments allow us to examine changes in Lat_ITCZ_, Lat_NHC_, Lat_SHC_ and Intensity_NHC_, and their driving mechanisms, by comparing the full impact of anthropogenic GHG under RCP8.5 (RCP8.5 minus historical simulations) with the direct radiative effect (amip4xCO_2_ minus amip), the indirect effect from uniform global SST warming of 4 K (amip4K minus amip), and the effect of spatially non-uniform warming patterns (amipFuture minus amip) (Fig. 1), as described next.

We find that both GHG increase and SST warming can force a poleward shift of Lat_SHC_ in this scenario, but indirect effects contribute more to this shift than the direct effect of GHG radiative forcing (Fig. 1A). Likewise, SST warming acts as a main driver of Lat_NHC_ expansion while GHG radiative forcing shows a large uncertainty in shifting Lat_NHC_ (Fig. 1B). Shifts in the HC edges in both hemispheres are thought to be related to changes in baroclinicity within the subtropics and mid-latitudes [30]. Static stability and vertical shear of the zonal winds are key factors influencing the baroclinicity of the subtropics and mid-latitudes [30–32]. The indirect effect decreases zonal wind shear in the subtropics and mid-latitudes of the Northern Hemisphere, which reduces atmospheric baroclinicity at the Lat_NHC_. This mechanism contributes to a stronger expansion of the Lat_NHC_ under the indirect effect rather than the direct effect of CO_2_ (Fig. S1). In contrast, the larger influence of the indirect effect on the Lat_SHC_ compared to the direct CO_2_ effect is likely due to increased static stability in the subtropics and mid-latitudes of the Southern Hemisphere, which reduces atmospheric baroclinicity at the Lat_SHC_ and enhances Lat_SHC_ expansion under the indirect effect (Fig. S2). The weaker direct effect of GHG forcing in the RCP8.5 experiment, compared to SST forcing in AMIP, is largely due to ozone recovery, which counteracts the influence of increasing GHG on the poleward expansion of the Hadley circulation [33,34]. Ocean warming, in addition to pushing HC edges poleward, can also move the ITCZ away from the equator (Fig. 1C), while the direct effects of GHG on Lat_ITCZ_ movement are weak and uncertain (Fig. 1C).

Unlike the consistent impact on HC and ITCZ position, the contribution of future SST warming to projected changes in Intensity_NHC_ is uncertain (Fig. 1D). Therefore, not only is future ocean warming the main driver of projected HC and ITCZ shifts (Fig. 1A–C), but also a source of uncertainty in the future changes in Intensity_NHC_ (Fig. 1D). This motivates us to further isolate the distinct contributions of warming in individual ocean basins to projected HC changes, in terms of Lat_NHC_, Lat_SHC_, Lat_ITCZ_ and Intensity_NHC_, and in particular establish which basins determine changes in HC and ITCZ positions and NHC intensity as described next.

### The roles of individual ocean basins in shaping HC characteristics in idealized experiments

Prior to disentangling the effects of regional ocean warming patterns on future HC changes, a suite of idealized experiments is conducted to compare differences in the responses of Lat_SHC_, Lat_NHC_, Lat_ITCZ_ and Intensity_NHC_ to the warming magnitudes of individual oceans (dashed rectangle boxes in Fig. S3A). This allows us to find the target basins that potentially affect future changes in the HC and ITCZ. Since the warming of the extratropical oceans is thought to have a minor impact on the HC [35], this study primarily focuses on testing the response of the HC to the warming of individual tropical oceans through idealized experiments in order to identify key regions of the tropical oceans where future warming could potentially shape future changes in the HC.

**Figure 2.**
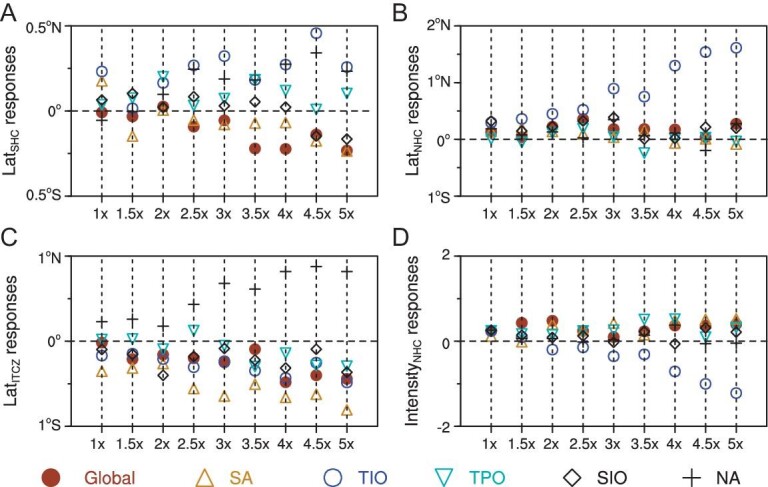
Idealized experiments to isolate the impacts of warming in individual ocean basins. The plot shows the response of (A) Lat_SHC_, (B) Lat_NHC_, (C) Lat_ITCZ_ and (D) Intensity_NHC_ to successively amplified historical warming patterns in each ocean basin. The numbers on the horizontal axis indicate the amplifying coefficients. Color markers at bottom denote particular ocean basins used in the experiments and SST warming pattern for each ocean basin is given in Fig. S3A. Note the strong response of HC to the warming of the tropical Indian Ocean, and the approximate compensation between the warmings in the South and North Atlantic.

Lat_SHC_ tends to extend poleward with increasing warming in the South Atlantic (SA) (Fig. 2A), while Lat_NHC_ extends poleward linearly with warming in the tropical Indian Ocean (TIO) (Fig. 2B). There is a remarkable shift of Lat_ITCZ_ toward the south pole with increasing ocean warming in TIO/SA, while Lat_ITCZ_ migrates linearly toward the equator as the tropical Northern Atlantic Ocean (NA) warms (Fig. 2C). SA and TIO warmings exert contrasting effects on the Intensity_NHC_. SA warming tends to increase Intensity_NHC_, whereas Intensity_NHC_ decreases linearly with TIO warming (Fig. 2D). Considering differences in the effects of warming in different basins on Lat_SHC_, Lat_NHC_, Lat_ITCZ_, Intensity_NHC_ in the idealized experiments (Fig. 2; Movie S1), we are further motivated to examine individual ocean warming-oriented impacts on the future HC and ITCZ. The response of the HC to increased warming in the South Indian Ocean (SIO) is very weak (Fig. 2). Moreover, a previous study [35] has shown that the extratropical oceans have a minimal influence on the HC. Therefore, experiments investigating the influence of future SIO warming on the HC were excluded. Although the HC is not particularly sensitive to the amplification of a fixed SST pattern in the tropical Pacific Ocean (TPO) (Fig. 2). However, given the considerable uncertainties in the projected future SST patterns in this region (Movie S2 and Movie S3), the TPO is also chosen as a candidate basin to assess the impact of future warming of the TPO on the HC. Therefore, four ocean basins (TIO, SA, TPO and NA) are used to investigate how future HC and ITCZ would respond to regional ocean warming patterns under 1.5°C, 2°C and 3°C temperature thresholds above the pre-industrial level.

### The Hadley cell and ITCZ responses to global and regional SST warming patterns under different future temperature thresholds

Considering that the robust future HC changes (weakening and expanding HC) and uncertainty in its future projection are both largely related to future SST warming patterns (Fig. 1) [28], what exactly is the impact of future ocean warming on the HC? Does it determine the robust changes or the uncertainty in the future HC response? To address this, we conducted large ensemble perturbation experiments on future global ocean warming and four regional ocean warming scenarios. Through comparative analyses of the HC responses to global and four regional ocean warming scenarios, we aim to identify the key ocean basins that determine the robust HC response and the sources of uncertainty in the HC response.

In contrast to the approach wherein fixed SST patterns were amplified in the idealized experiments (Fig. S3A), imposing projected global and regional SST patterns with three temperature thresholds shows a large inter-model spread (Movie S2 and Movie S3). Nevertheless, we impose all these available future SST warming patterns to force CAM4, in order to highlight the robust responses of the future HC (Lat_SHC_, Lat_NHC_, Lat_ITCZ_, Intensity_NHC_) and the uncertainties associated with regional ocean warming patterns (Movie S4 and Movie S5).

Before analyzing the contributions of individual ocean basins’ warming to changes in HC characteristics (Lat_SHC_, Lat_NHC_, Lat_ITCZ_, Intensity_NHC_), the spatial structure of the HC response to the warming of each ocean is examined. The reason is that future changes in the HC characteristics, as defined in the Supplementary Data, depend on the spatial changes in HC. We show that the spatial response of the Hadley cells to TIO warming projected by different models, as well as to the warming of SA and NA, is robust (Movie S4B, C, D and S4G, H, I; Movie S5D, B, C). In particular, the TIO warming can force a similar spatial pattern of HC changes as projected by previous studies (Movie S4B, G and Movie S5D) [36–38]. In contrast, the inter-model spread of global and TPO warming introduce a considerable uncertainty in the spatial distribution of the HC response (Movie S4A, F and S4E, J; Movie S5A, E). These spatially different responses of the HC to warming of the individual ocean basins can have different effects on the HC characteristics, as shown in the following section.

**Figure 3.**
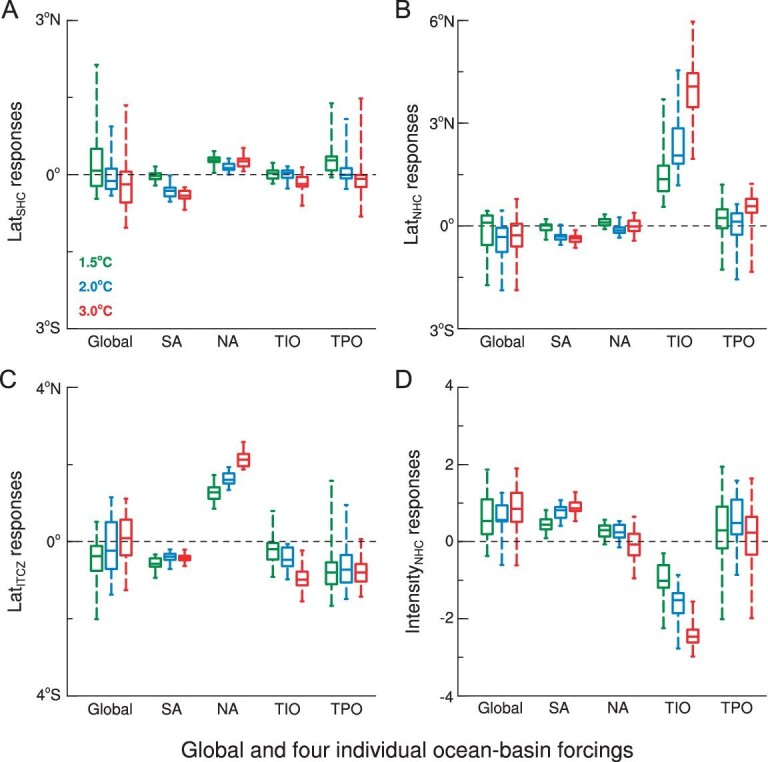
The Hadley cell and ITCZ responses to global and four individual ocean-basin forcings under different warming thresholds. Box-whisker plots show the minimum, 25th, 50th, 75th percentiles and maximum values for (A) Lat_SHC_, (B) Lat_NHC_, (C) Lat_ITCZ_ and (D) Intensity_NHC_ responses (relative to the reference period 1986–2005) to five ocean basin forcings. Three temperature thresholds above the pre-industrial are used: 1.5°C (green box-whisker), 2°C (blue) and 3°C (red). We find opposing effects of SA and NA on the shift of Hadley cell in the Southern Hemisphere, a strong effect of TIO on poleward shift of the Hadley cell in the Northern Hemisphere, opposite roles of NA and SA in ITCZ shift, distinct impacts of SA and TIO on the intensity of the Hadley cell in the Northern Hemisphere, and the uncertainty in the Hadley cell and ITCZ response mainly arising from TPO forcing.

Forcing by the global SST pattern induces large uncertainties for all HC characteristics (Lat_SHC_, Lat_NHC_, Lat_ITCZ_, Intensity_NHC_). Analyzing the response to specific patterns in four ocean basins, we can pinpoint the robust characteristics, but also the origin of the spread between model responses (Fig. 3). We find distinct responses from four separate ocean basins’ forcings that are responsible for robust responses and uncertainties in HC characteristics (Fig. 3). SA forces a weak poleward shift of Lat_SHC_ as temperature thresholds rise (Fig. 3A). In contrast, poleward migration of Lat_NHC_ increases linearly with the warming of the TIO (Fig. 3B), while SA forcings act a weak role in shrinking Lat_NHC_ (Fig. 3B). TIO forcing dominates ITCZ shift off the equator and SA forcing contributes secondly to such a shift (Fig. 3C). In contrast, NA warming moves the ITCZ toward the equator and the equatorward shift increases with a rise in the temperature threshold (Fig. 3C). The opposite effects of TIO and SA forcings on Intensity_NHC_ are also evident. Intensity_NHC_ decreases as TIO warms and Intensity_NHC_ increases as SA warms (Fig. 3D). In addition to the robust response to these three separate ocean warmings (TIO, SA, NA), TPO forcing gives rise to the major uncertainty for all HC characteristics Lat_SHC_, Lat_NHC_, Lat_ITCZ_ and Intensity_NHC_ (Fig. 3).

### Mechanisms explaining the robust responses of HC and uncertainties under different temperature thresholds

**Figure 4.**
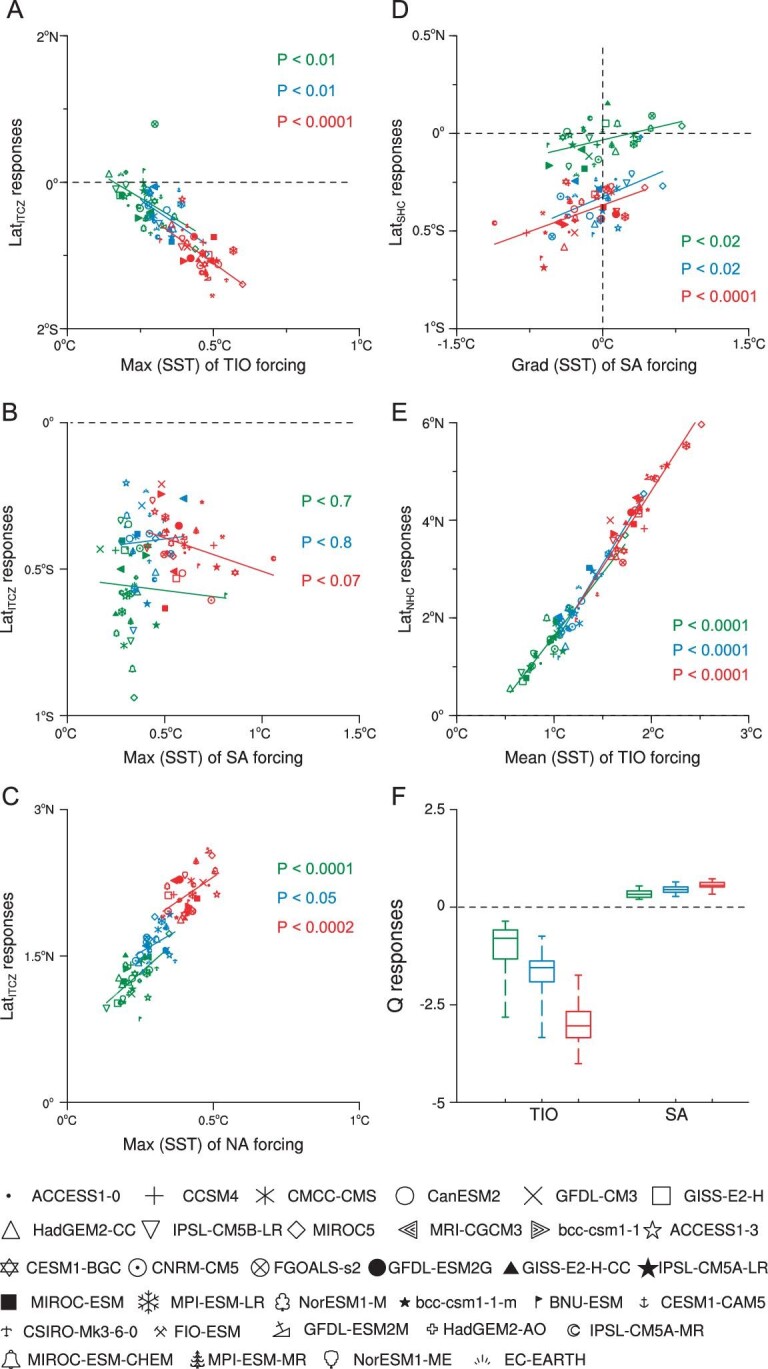
Isolating the mechanisms of Lat_ITCZ_, Lat_SHC_, Lat_NHC_ and Intensity_NHC_ responses to future regional SST warming patterns. The same temperature thresholds of 1.5°C, 2°C and 3°C as in Fig. 3 are used. Left panels represent the latitude of ITCZ migrations (Y axis) against interhemispheric thermal contrast (on the horizontal axis) arising from separate ocean forcings defined as (A) maximum future TIO heating, (B) maximum future SA warming, (C) maximum future NA warming. Right panels (from top to bottom) describe (D) meridional shift of Lat_SHC_ against meridional SST contrast under the SA forcing, (E) meridional shift of Lat_NHC_ against rising mean TIO SST, (F) contrasting effects of SA and TIO on Intensity_NHC_ through opposing tropical diabatic heating/cooling (Q) responses to SA and TIO forcings. The markers at bottom indicate the CMIP5 models whose future projections of ocean warming patterns under three temperature thresholds are used to force CAM4. Text labels for P-value in each panel indicate the significance level of the linear regression coefficient between the two variables.

Meridional shifts of Lat_ITCZ_ are regulated by inter-hemispheric differential heating [39,40], i.e. ITCZ tends to migrate toward a warmer hemisphere. Taking into account that the climatological Lat_ITCZ_ in boreal winter is located south of the equator, TIO warming with maximum oceanic heating is confined to a region south of the equator and thus causes a southward shift of Lat_ITCZ_ away from the equator (Fig. 4A). Maximum warming in SA is not sufficient to cause a significant southward shift in the ITCZ (Fig. 4B), which is consistent with a weak Lat_ITCZ_ shift under SA forcing (Fig. 3C). In contrast, the maximum warming in NA alters the thermal contrast between the two hemispheres (Fig. 4C), which leads to a significant equatorward shift of the ITCZ (Fig. 3C).

Mean SST warming and SST warming patterns are considered as two main drivers of HC expansion that have been observed since 1979 [41]. Their dominant control of future Lat_SHC_ and Lat_NHC_ under the three temperature thresholds used can be understood as follows. The main reason for the southward expansion of Lat_SHC_ is the weakening of the equator-to-pole temperature gradient due to the non-uniform SA warming (Fig. 4D), whereas the mean SST warming in SA contributes little to the poleward expansion of Lat_SHC_ (Fig. S4). Instead, it is the overwhelming effect of rising mean SST in TIO that causes a significant expansion of Lat_NHC_ (Fig. 4E), although the increase in the equator-to-pole gradient due to a non-uniform warming in the TIO contributes to a reduction of Lat_NHC_. In addition, future warming of the TIO is expected to increase static stability in the Northern Hemisphere subtropics, reducing atmospheric baroclinicity at the Lat_NHC_ and thus pushing the Lat_NHC_ poleward (Fig. S5). This mechanism also serves as an alternative explanation for the expansion of the Lat_NHC_ driven by future TIO warming.

Considering that the HC is thermally direct, we can use tropical diabatic heating/cooling (Q), as a more fundamental physical variable than atmospheric static stability ($\frac{{\partial \theta }}{{\partial p}}$), to understand the thermal effect of future regional ocean warming on Intensity_NHC_ (the definition of Q and its advantages over $\frac{{\partial \theta }}{{\partial p}}$ are given in Supplementary Materials; Fig. S6; Movies S6–S9). TIO warming and SA warming have distinct thermal effects on the Intensity_NHC_ for the three warming thresholds considered. The diabatic cooling of the tropical atmosphere due to weakened vertical motion (i.e. dynamic cooling) in response to future TIO warming reduces Intensity_NHC_ (Fig. 4F), while strengthening of the Intensity_NHC_ in the future is due to the diabatic heating response to SA warming (Fig. 4F). Meanwhile, the Q perspective helps (1) explain why TIO warming is the most important driver for future HC compared with other ocean basins and (2) identify the source of uncertainty in HC responses by comparing the response of the atmospheric thermodynamic structure to heating of global and individual ocean basins (Movie S7 and Movie S8). Within the HC domain, the tropical atmosphere's thermodynamic structure exhibits a more pronounced response to future warming of the TIO compared to other ocean basins. This likely explains why TIO warming is the main driver of future HC changes (Movie S7 and Movie S8). The high sensitivity of the spatial distribution of atmospheric thermodynamic structure to future TPO warming patterns explains the uncertainty ranges for all HC characteristics (Lat_SHC_, Lat_NHC_, Lat_ITCZ_ and Intensity_NHC_) (Movie S7 and Movie S8).

## CONCLUSIONS AND IMPLICATIONS

In summary, we have examined the relative impacts of CO_2_ radiative forcing and regional ocean warming patterns on future HC changes. Our work highlights regional ocean controls of both the response and uncertainty of future HC changes. i.e. ocean warming in three ocean regions (TIO, SA, NA) determines the robust responses of Lat_SHC_, Lat_NHC_, Lat_ITCZ_, Intensity_NHC_, and ocean warming in TPO controls the uncertainty range in the main HC characteristics. Finally, our study highlights the enhanced sensitivity of the HC and ITCZ to warming in the tropical Indian Ocean. This is also consistent with previous studies highlighting the role of TIO warming in atmospheric teleconnections [42,43]. In contrast to the different responses of HC to the warming in four separate ocean basins, precipitation generally follows the ‘warm gets wet’ response in those ocean basins (Fig. S7).

We note that a recent study [44] has reconciled the inconsistency in long-term HC trends between atmospheric reanalysis data and climate models during the observational period. This study highlights an artificially enhanced diabatic heating in the reanalyzed data that leads to HC strengthening in the reanalysis, which is opposite to climate model simulations and the observed precipitation data. To address this issue, we superimpose the observed SST trends in TIO for two periods (1951–2000 and 1979–2017) on the climatological mean monthly SST in CAM4 (Fig. S8A, B). The results show that the amplitude of the observed TIO warming is not sufficient to cause the weakening of the HC (c.f. Fig. S8C, D and Fig. 2D), but it can lead to a significant poleward expansion of Lat_NHC_ during the two observational periods (Fig. S8C, D). Additionally, changes in the meridional surface temperature gradient of the Pacific Ocean have provided key insights into the expansion of the HC since the 1980s [45]. By integrating previous findings [41,45] with the results of our study, there may be two distinct mechanisms through which warming in the Pacific Ocean and the TIO modulates the observed HC shift. The Pacific impacts on the poleward expansion of the HC through changes in the SST meridional gradients due to spatially non-uniform ocean warming, while the TIO causes the poleward shift through increase in mean SST (see Fig. 4E).

Thus, our investigation provides insights into the future response of the Hadley cell, and more generally of atmospheric tropical meridional circulation, as related to changes in the SST patterns, particularly over the Indian Ocean. By examining the impact of seasonally-dependent warming in the TIO on HC (Fig. S9) and comparing it with the HC's response to warming in other ocean basins (TPO, NA, SA) (Figs S10–S12), we demonstrate that future warming of the TIO is the primary driver of HC weakening and poleward expansion. The overwhelming role that the future warming of the tropical Indian Ocean plays in the Hadley circulation provides a potential scientific basis for monitoring and managing climate risks associated with future changes in the Hadley circulation. At the same time, we have confirmed that warming of the tropical Pacific Ocean is the main source of uncertainty in the projections for the Hadley circulation, which provides new directions for improving the performance of Earth system models. We note that the HC edges (Lat_NHC_, Lat_SHC_) and intensity (Intensity_NHC_, Intensity_SHC_) in each hemisphere display significant seasonality in response to future seasonally-dependent warming in the TIO (Fig. S9). This factor, along with the response of the Lat_ITCZ_ to seasonally-dependent warming in different ocean basins (Fig. S13), warrants further investigation.

Our work not only clarifies the regional oceanic control of robust responses and uncertainties in future changes of the Hadley circulation using large ensemble experiments, but also extends the experimental framework of CLIVAR (Climate and Ocean: Variability, Predictability and Change), a core project of the World Climate Research Programme (WCRP). The CLIVAR experiments are limited to idealized scenarios with linear warming trends and internal variability of sea surface temperature (e.g. AMO, PDO) within the observation period (https://gmao.gsfc.nasa.gov/research/clivar_drought_wg/), without considering the impact of future ocean warming. Our study addresses this limitation and provides a more comprehensive approach to projecting climate change.

## DATA AND METHODS

1. To evaluate the relative impact of direct radiation forcing versus indirect SST warming on future changes in the HC, we utilize the currently available CMIP5 experiments. These experiments encompass four AMIP-type simulations (e.g. amip, amip4K, amip4 × CO_2_, amipFuture), along with historical climate simulations and future climate projections under the RCP8.5 scenario (Table S1). We show that both the robust future HC changes and the uncertainty in the future projection are largely related to future SST warming.
2. To further clarify the impact of regional ocean warming on robust future HC changes and uncertainty in future projections, a series of idealized SST forcing (Fig. S3) experiments were conducted using CAM4 to test and assess sensitivity of Lat_SHC_, Lat_NHC_, Lat_ITCZ_ and Intensity_NHC_ to warming in individual ocean basins and to identify important basins that could potentially impact future HC behaviors (e.g. robust changes and uncertainty).
3. Four ocean basins (TIO, SA, TPO and NA) were identified using the idealized experiment, and large ensemble experiments were conducted with four separate oceans and global future SST forcings (Movie S2 and Movie S3) to investigate how future HC and ITCZ will respond to regional ocean warming patterns at temperature thresholds of 1.5°C, 2°C and 3°C above pre-industrial levels.

The details of data, experimental designs and metrics are available in the Supplementary Materials.

## Supplementary Material

nwae375_Supplemental_File

## DATA AND MATERIALS AVAILABILITY

All the CMIP5 experiments listed in the Table S1 are available: https://www.ipcc-data.org/sim/gcm_monthly/AR5/Reference-Archive.html. Access to the data used for the plots in this study is available online: https://doi.org/10.5061/dryad.nvx0k6f0z. Additional results of the SST perturbation simulations are available from the corresponding author: Dr. Yong Sun, yongsun@itpcas.ac.cn. The CESM code can be obtained from the following website: https://www.cesm.ucar.edu/models/releases. ERSST V3b is available: https://psl.noaa.gov/data/gridded/data.noaa.ersst.v3.html.
