# Supplementary material for: Tropical Indian Ocean drives Hadley circulation change in a warming climate
Source: Natl Sci Rev. 2024 Oct 22;12(1):nwae375. doi: 10.1093/nsr/nwae375 (PMC11702687; doi:10.1093/nsr/nwae375)
Supplement: nwae375_Supplemental_File [file nwae375_supplemental_file.docx]

Supplementary Materials for

**Tropical Indian Ocean drives Hadley circulation change in a warming climate**

Yong Sun^1,2^, Gilles Ramstein^2^, Alexey V. Fedorov^3,4^, Lin Ding^1^, Bo Liu^5^

^1^State Key Laboratory of Tibetan Plateau Earth System, Environment and Resources (TPESER), Institute of Tibetan Plateau Research, Chinese Academy of Sciences, Beijing 100101, China

^2^Laboratoire des Sciences du Climat et de l'Environnement, LSCE/IPSL, CEA‐CNRS‐UVSQ, Université Paris‐Saclay, Gif‐sur‐Yvette 91191, France

^3^Department of Earth and Planetary Sciences, Yale University, New Haven, CT 06511, USA

^4^LOCEAN/IPSL, Sorbonne University, Paris 75005, France

^5^Plateau Atmosphere and Environment Key Laboratory of Sichuan Province, School of Atmospheric Sciences, Chengdu University of Information Technology, Chengdu 610225, China

Corresponding author : Yong Sun

Email: yongsun@itpcas.ac.cn

**Supplementary Materials for this paper include the following:**

Data, Experimental designs, and Metrics

Figs. S1 to S13

Table S1

Movies S1 to S9

References (46–57)

**Data, Experimental designs, and Metrics**

**CMIP5 experiments and SSTA forcing experiments**

To assess the relative importance of direct radiation forcing and indirect SST warming to the future changes in HC, we retrieve the currently available CMIP5 experiments, including four AMIP-type’s simulations (e.g., amip, amip4K, amip4$\times$CO_2_, amipFuture), historical climate simulation and future climate projection in RCP8.5 scenario (Table S1). The relative importance of direct radiative effect and indirect effect through SST feedback on HC changes in RCP8.5 scenario compared to those of the present day (1986–2005) in the historical simulation can be evaluated as the sum of differences between amip4$\times$CO_2_ and amip, amip4K and amip, amipFuture and amip. One issue should be kept in mind: these AMIP-type’s experiments could not exactly represent the realistic responses of HC to anthropogenic future ocean warming, albeit such a decomposition is helpful to assess the relative contribution of direct CO_2_ radiation and SST changes to future projection of HC. For example, it lacks spatial inhomogeneity of SST forcing in the AMIP4K experiment, while CMIP3 rather than CMIP5 projected ocean warming patterns are prescribed in these amipFuture experiments. This deficiency inspires us to re-examine the realistic responses of HC to future ocean warming patterns by prescribing projected SST patterns of multi CMIP5 models to force a same climate model.

Because the tropics are a region of strong air-sea interactions, one intuitively assumes that coupled atmosphere-ocean models (AOGCMs) could be more appropriate for studying the response of tropical atmospheric circulation to ocean warming. However, this is not always the case, and it has been shown that ocean-atmosphere coupling can obscure the system’s response to anthropogenic forcing by modifying, sometimes greatly, internal climate variability in coupled GCMs under different warming scenarios [46]. Therefore, in this study, we use AGCMs to examine the HC response to ocean warming.

Two major scaling theories are commonly used to explain HC changes [47,48]. However, the intricate linkages within the complex climate system pose a challenge for conducting a causal analysis of HC changes relying solely on the physical parameters of these theories [49]. Since SST serves as the primary boundary condition prescribed in AGCM experiments, we therefore use an AGCM to perform a large ensemble of SST perturbation experiments in which the SST served as the only external forcing change, which allows us to find the critical drivers of future HC changes and to give a mechanistic explanation of the changes.

Finally, the increase in mean SST and changes in the meridional SST gradient due to spatially non-uniform ocean warming are two important physical processes that are used to explain the observed expansion of the HC [41]. The large ensemble of future SST perturbation experiments under 1.5$℃$, 2$℃$ and 3$℃$ above the pre-industrial level, which we conduct with prescribed SST forcings, considers both aspects of SST distribution that can potentially influence future changes in HC, allowing for an analysis of the forcing and the response.

**Model description**

The climate model used in this study is Community Atmosphere Model version 4 (CAM4) that is a standalone atmospheric component of Community Earth System Model version 1.2.1 (CESM1.2.1) with a horizontal grid of 1.9° in latitude by 2.5° in longitude and 26 vertical levels [50].

**Idealized SSTA sensitivity experiments**

To identify candidate ocean basins that can potentially influence the HC in the future, a series of SSTA sensitivity experiments (i.e., idealized experiments) are conducted on the basis of present-day climate. The present-day climate simulation is a built-in model experiment (F2000) with climate parameters fixed in year 2000. An idealized sea surface warming pattern (i.e., SSTA), constructed from the first leading mode of ERSST version 3 via Empirical orthogonal function (EOF) spanning the period 1900-2006 (Fig.S3A) is used; we successively multiply this pattern by coefficients ranging from 1 to 5 with an increment of 0.5. These idealized simulations are superimposed over the climatological annual cycle SST of the present climate simulation. This means that these amplified global and regional ocean basin SST warming patterns serve as prescribed SSTA forcings in the idealized experiments which are used to investigate the sensitivity of HC to increased magnitudes of global and regional SST forcings (Movie S1). There are 54 idealized experiments that consist of six test ocean basins including the global ocean (90°S–90°N, 0°–360°; Global) tropical Pacific Ocean (30°S–30°N, 160°–285°E; TPO), tropical Indian Ocean (20°S–25°N, 40°–105°E; TIO), Southern Indian Ocean (60°–30°S, 30°–210°E; SIO), Southern Atlantic Ocean (60°S–0°, 60°W–30°E; SA), Northern Atlantic Ocean (0°–30°N, 75°W–10°W; NA) (Fig.S3A). In our experiment, we specifically focus on the impact of tropical ocean warming on HC, as previous study [35] has indicated that warming outside the tropics generally has little effect on HC. Parts of the Southern Ocean are included in the idealized experiment due to substantial warming there that may affect HC ([42]; Fig.S3A).

**Future SSTA forcings under 1.5**$\mathbf{℃}$**, 2**$\mathbf{℃}$ **and 3**$\mathbf{℃}$ **target experiments**

To disentangle the forcing roles of regional SST future warming due to increase of Greenhouse gases, and to elucidate which ocean basin forcing is the source of uncertainty in projection of HC in response to future SST warming, the experimental configurations in the future SSTA forcings experiments are kept the same as those in the idealized experiments, except that future SST warming patterns are prescribed to force CAM4. Future SST warming patterns relative to the reference period (1986–2005, historical simulation) are obtained under temperature thresholds of 1.5$℃$, 2$℃$ and 3$℃$ (i.e., global average of surface air temperature (SAT) in boreal winter gets to 1.5$℃$, 2$℃$ and 3$℃$ above the pre-industrial level). Movie S2 lists future SST warming patterns as projected SAT rises in RCP4.5 scenario reaching 1.5$℃$ (31 models) and 2$℃$ (23 models) above the pre-industrial level. Movie S3 shows the future SST warming patterns of 32 climate models under target of 3$℃$ in RCP8.5 scenario. All global and four separate ocean basin SST warming patterns under targets of 1.5$℃$, 2$℃$ and 3$℃$ are superimposed over the climatological annual cycle SST of present climate simulation (F2000) to force CAM4. Consequently, 430 experiments are needed to run.

Overall, 484 sensitivity experiments (54 idealized experiments and 430 under three temperature target experiments) are conducted. Each sensitivity experiment is initialized from the 21st year of the F2000 simulation with the constructed SSTA forcings for the idealized experiments and 1.5℃, 2℃ and 3℃ target experiments superimposed over the CAM4. We run 50 model years for the F2000 simulation and 31 model years for each sensitivity experiment. The last 30 years of each simulation are used for comparison with the F2000 simulation. Each simulation has monthly output, except for the daily frequency output of several variables that are required to calculate the diabatic heating.

As this work was initiated before the release of CMIP6. Considering the relatively small improvements in model development and prescribed forcings between CMIP5 and CMIP6, previous studies have shown similar performance in simulating HC in both datasets [5,6,51]. Therefore, the conclusions resulting from the analysis of the existing CMIP5 experiments and the large ensemble simulations based on the CMIP5 projected future SST warming patterns remain robust and valid.

**Metrics for Lat_ITCZ_, Lat_NHC_, Lat_SHC_, Intensity_NHC_ and Q**

Precipitation centroid was used to measure the position of the ITCZ (Lat_ITCZ_) [52].

MSF, calculated as vertical integral of zonal mean meridional wind over the troposphere, is a conventional metric of the HC [53,54]. The formula is expressed as:

$\psi\left( \emptyset,p \right)=\frac{2\pi a\cos\emptyset}{g}\int_{0}^{p} v\left( \emptyset,p \right)dp$

where π is a circular constant, a is the Earth’s radius, $\emptyset$ is latitude, g is the gravity acceleration, p is pressure, and v is the meridional velocity.

Lat_SHC_ and Lat_NHC_ are the latitudinal positions where the zero value of MSF at 500 hPa occurs in the subtropics of Southern and Northern Hemispheres, respectively [3]. The Intensity_NHC_ is measured by the maximal MSF between 30°S and 30°N [31,53,54].

Diabatic heating/cooling was calculated using daily variables as a residual of thermodynamic equation [54,55]

$$Q=\frac{\Delta T}{\Delta t}+\overline{\boldsymbol{V}}\cdot\nabla\overline{T}+\left( p/{p_{0}} \right)^{\left( R/{C_{p}} \right)} \overline{\omega}\frac{\partial\theta}{\partial p}+\left( p/{p_{0}} \right)^{\left( R/{C_{p}} \right)}\left[ \nabla\cdot\overline{\boldsymbol{V}^{'}\theta^{'}}+\frac{\partial\overline{\left( \omega^{'}\theta^{'} \right)}}{\partial p} \right]$$

where overbars indicate monthly averages and primes indicate the departure of daily variables from the monthly average; T is air temperature, ***V*** is horizonal wind vector, $\theta$ is potential temperature, $\omega$ is vertical velocity, R is the gas constant and $C_{p}$ is specific heat capacity at constant pressure.

We use diabatic heating/cooling because of several major factors as summarized below:

1. These variables describe large-scale HC features [31], since the tropical diabatic heating and subtropical cooling processes are consistent with the upward and downward motion of the HC in the NCEP-DOE 2 reanalysis (Fig.S6A), the F2000 simulation (Fig.S6B), and the SST perturbation experiment under three temperature thresholds (Movie S6). It is noteworthy that climate models often overestimate diabatic heating as compared to reanalyses [54]. This overestimation is evident in our study as well (Fig.S6).
2. They allow us to explore the contrasting thermodynamic structures of the tropical atmosphere in responses to global and regional basins forcings under three temperature thresholds (Movie S7 and Movie S8), because diabatic heating and cooling processes characterize the rising and sinking of tropical atmospheric circulation (Movie S6), and at the same time their changes are spatially consistent with the anomalous rising and sinking motions of HC in response to global and regional basins forcings (Movie S7 and Movie S8) [31].
3. These variables are more fundamental for the tropical circulation than the static stability of the atmosphere, which is often used to explain HC changes [30-32]. In the tropics, the thermodynamic equation can be simplified to the following form: $Q\approx\left( p/{p_{0}} \right)^{\left( R/{C_{p}} \right)} \overline{\omega}\frac{\partial\theta}{\partial p}$ [31]. This underscores the advantage of diabatic heating ($Q$) over atmospheric static stability ($\frac{\partial\theta}{\partial p}$) in explaining HC strength changes [31,56], because $\frac{\partial\theta}{\partial p}$ is only one of several factors influencing diabatic processes Q, as manifested in the simplified thermodynamic equation [57]. For instance, TIO warming and SA warming tend to force an increase in the static stability of the atmosphere (Movie S9), so that the HC is supposed to weaken with TIO warming and SA warming, but in fact TIO warming and SA warming show contrasting effects on the strength of HC (Intensity_NHC_) (Fig.3D). Since the maximum diabatic heating occurs in the mid-troposphere [54,55], it is calculated at 400hPa using the residual of thermodynamic equation (averaged over 10°S ~0° for SA forcing and 5°S ~5°N for TIO forcing) [57] to explore the contrasting thermodynamic responses of Intensity_NHC_ to SA forcing and TIO forcing under three temperature thresholds (Fig.3D). The changes in Q are decomposed into thermodynamic contributions due to changes in static stability (i.e., thermodynamic warming or cooling) and dynamic contributions due to changes in vertical motion (i.e., dynamic warming or cooling). This decomposition helps to assess the relative roles of the thermodynamic and dynamical components in shaping the response of the HC to future regional SST warming patterns [31, 56].
4. They help identify the causes of uncertainty in HC responses to future SST warming patterns (Movie S7A, E; Movie S7F, J and Movie S8A, E).

**

**Fig.S1** scatter plot showing the relationship between Lat_NHC_/Lat_SHC_ and Northern Hemisphere (NH)/Southern Hemisphere (SH) subtropical zonal wind shear (U500–U850, as defined by [31]) under 4xCO₂ forcing (left panel) and SST changes (right panel). The p-value denotes the significance of the linear regression coefficient between the two variables.

**

**Fig.S2** As in Fig.S1, but showing the relationship between Lat_NHC_/Lat_SHC_ and NH/SH subtropical static stability (defined as the difference in potential temperature between 400 and 850 hPa, averaged between 25° and 50°, following [32]) in response to 4xCO₂ forcing and SST changes.


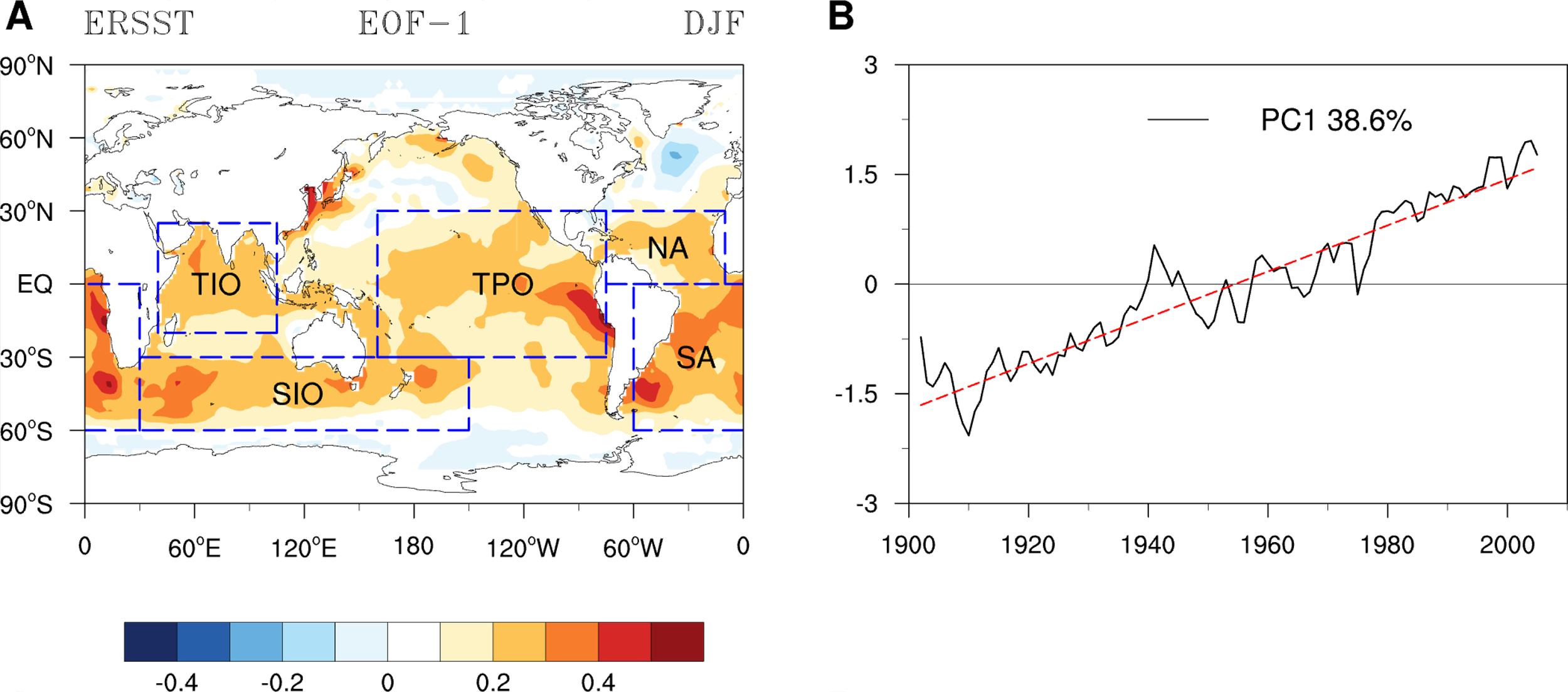


**Fig.S3** Global and regional SST warming patterns in boreal winter (December-January-February, DJF) used to force CAM4 in the idealized experiments and obtained from EOF analysis applied to ERSST spanning the period 1900–2006. Panel left is first leading EOF model (EOF-1) associated with the first principal components (PC1) and explained variances (38.6%) are on the panel right. The EOF-1 globally and regionally amplifies the magnitudes over tropical Pacific Ocean (30°S–30°N, 160°–285°E; TPO), tropical Indian Ocean (20°S–25°N, 40°–105°E; TIO), Southern Indian Ocean (60°–30°S, 30°–210°E; SIO), Southern Atlantic Ocean (60°S–0°, 60°W–30°E; SA), Northern Atlantic Ocean (0°–30°N, 75°W–10°W; NA), by multiplying a coefficient from 1 to 5 by an increment of 0.5. These amplified SST warming patterns are SSTA forcings in the idealized experiments which are superimposed over the present climate conditions.


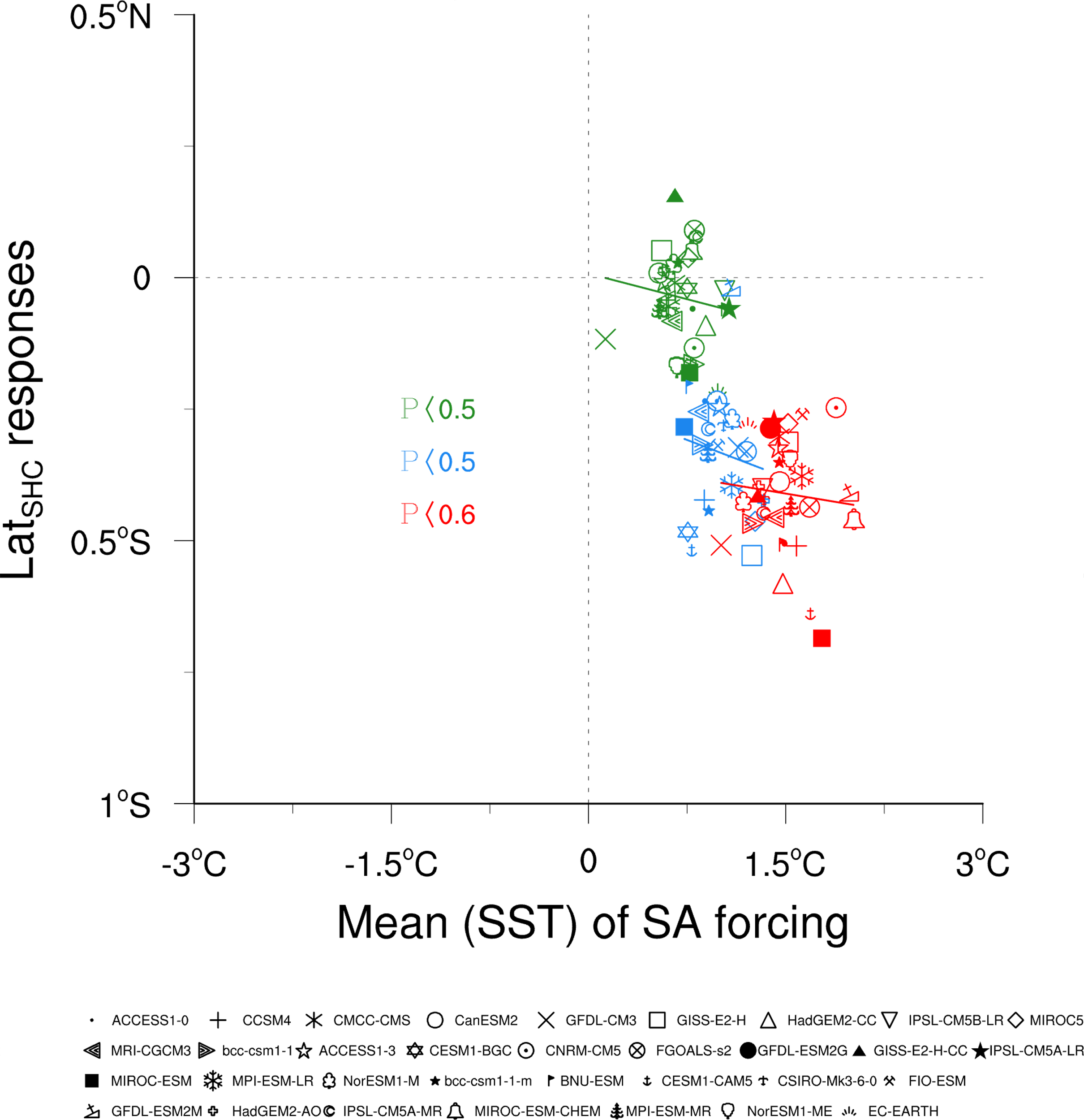


**Fig.S4** As Fig.4E, but showing Lat_SHC_ responses against mean SSTA of SA forcing under three temperature thresholds. The p-value indicates the significance level of the linear regression coefficient between the two variables.

**

**Fig.S5** Meridional shift of Lat_NHC_ against NH subtropical static stability (defined as the difference in potential temperature between 400 and 850 hPa, averaged between 25° and 50° N, following [32]) under the TIO forcing at 1.5$℃$, 2$℃$, and 3$℃$ warming thresholds. The p-value indicates the significance level of the linear regression coefficient between the two variables.


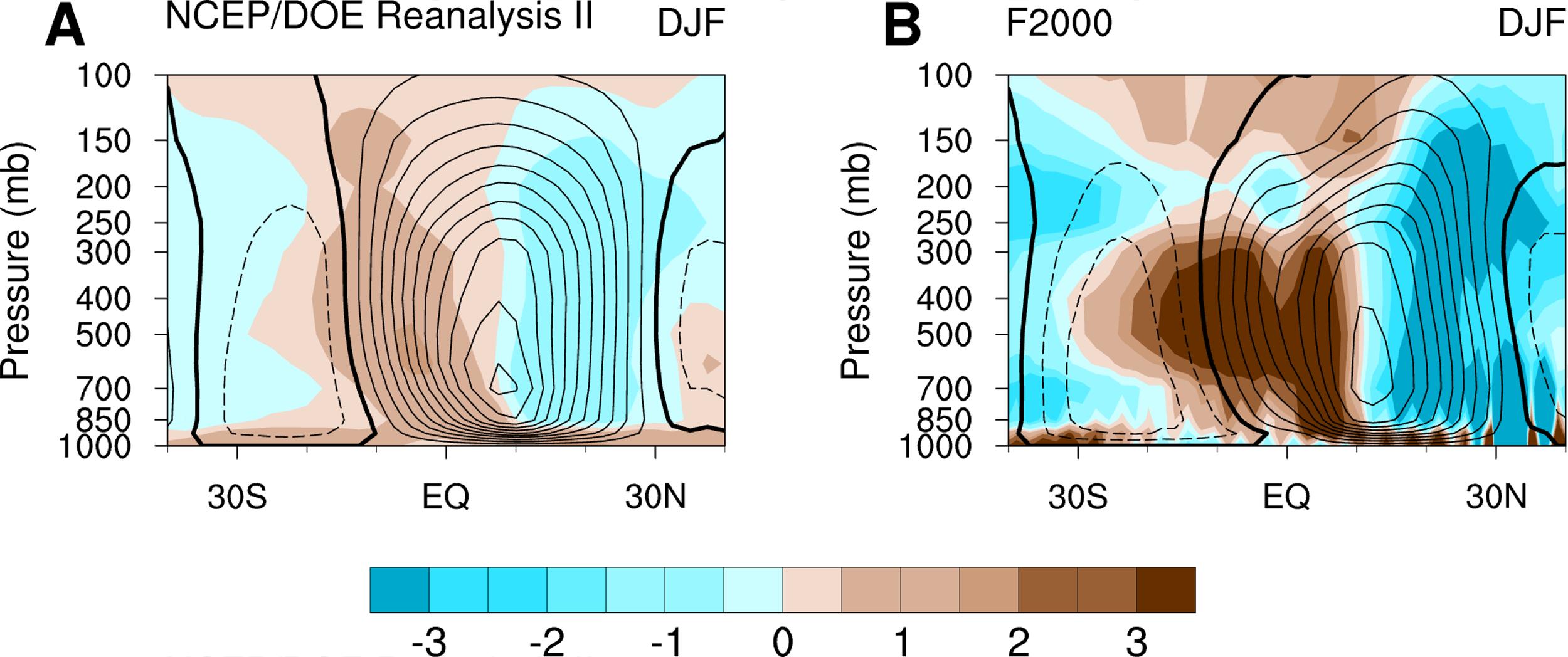


**Fig.S6** Spatial distribution of the mean state of tropical diabatic heating and subtropical cooling in boreal winter (shaded, K/day) compared to the mean state of the rising and sinking of HC, represented by the MSF (contours: 10^10^ kg$\cdot$s^-1^) for **A** NCEP-DOE Reanalysis 2 (1979-2008) and **B** F2000 simulation.


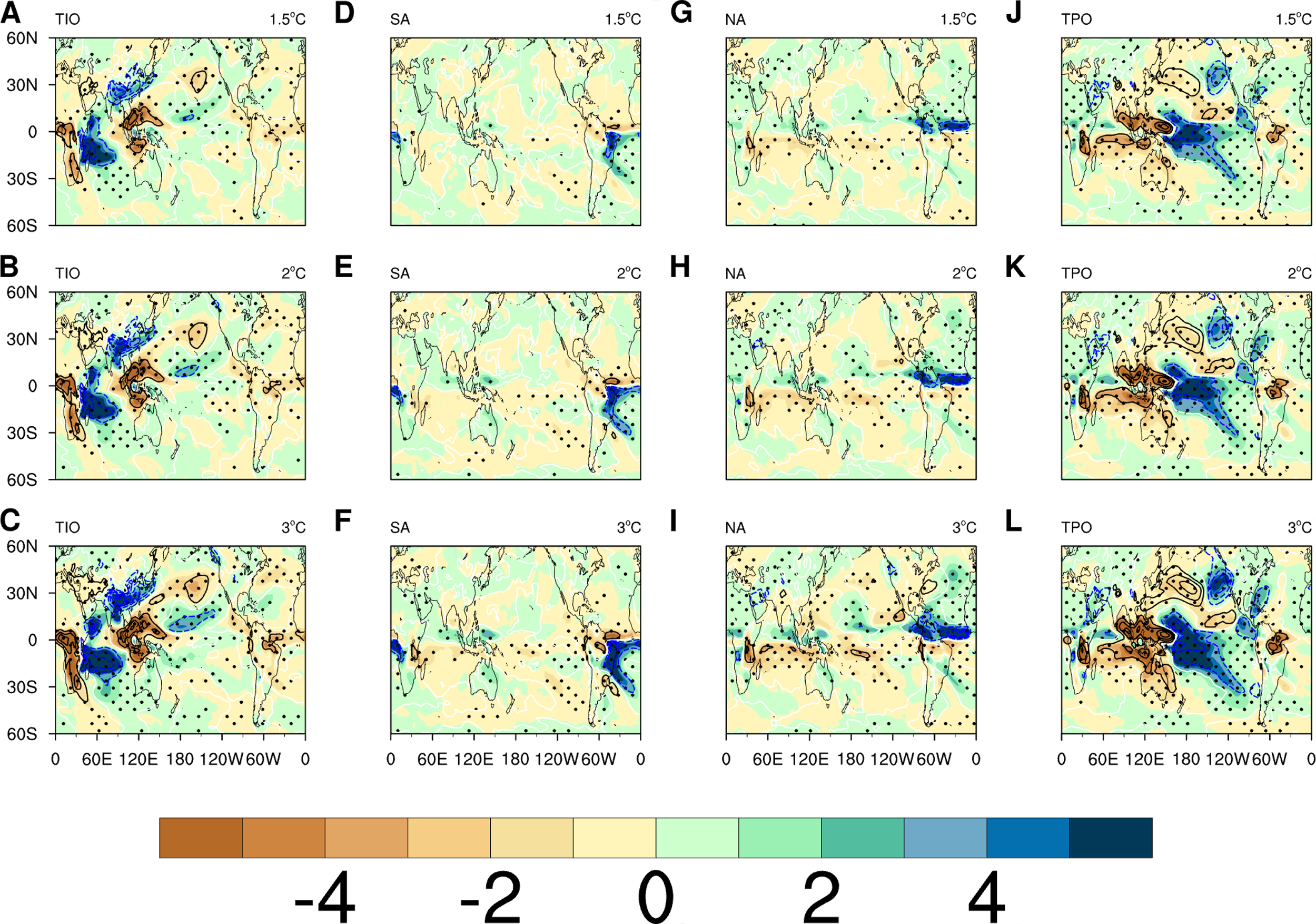


**Fig.S7** Large ensemble mean of precipitation (shading: mm/day) and vertical velocity (contours: 10^-2^ Pa/s) in response to warming of individual ocean basins (TIO, SA, NA and TPO) under temperature thresholds of $1.5℃$, 2$℃$and 3$℃$ above the pre-industrial level. The white contours in each diagram are used to distinguish between anomalous upward motion (blue dashed) and downward motion (black solid). Dotted areas indicate precipitation responses with significance above 1%.

**Fig.S8** The observed ocean warming trends for two periods: **A** 1951-2000 ($℃$/50 years) and **B** 1979-2017 ($℃$/39 years). The response of HC (10^10^ kg/s) to the observed TIO warming during these two periods when prescribed in CAM4 is shown in **C** and **D.** Significance of each figure above 1% is dotted.


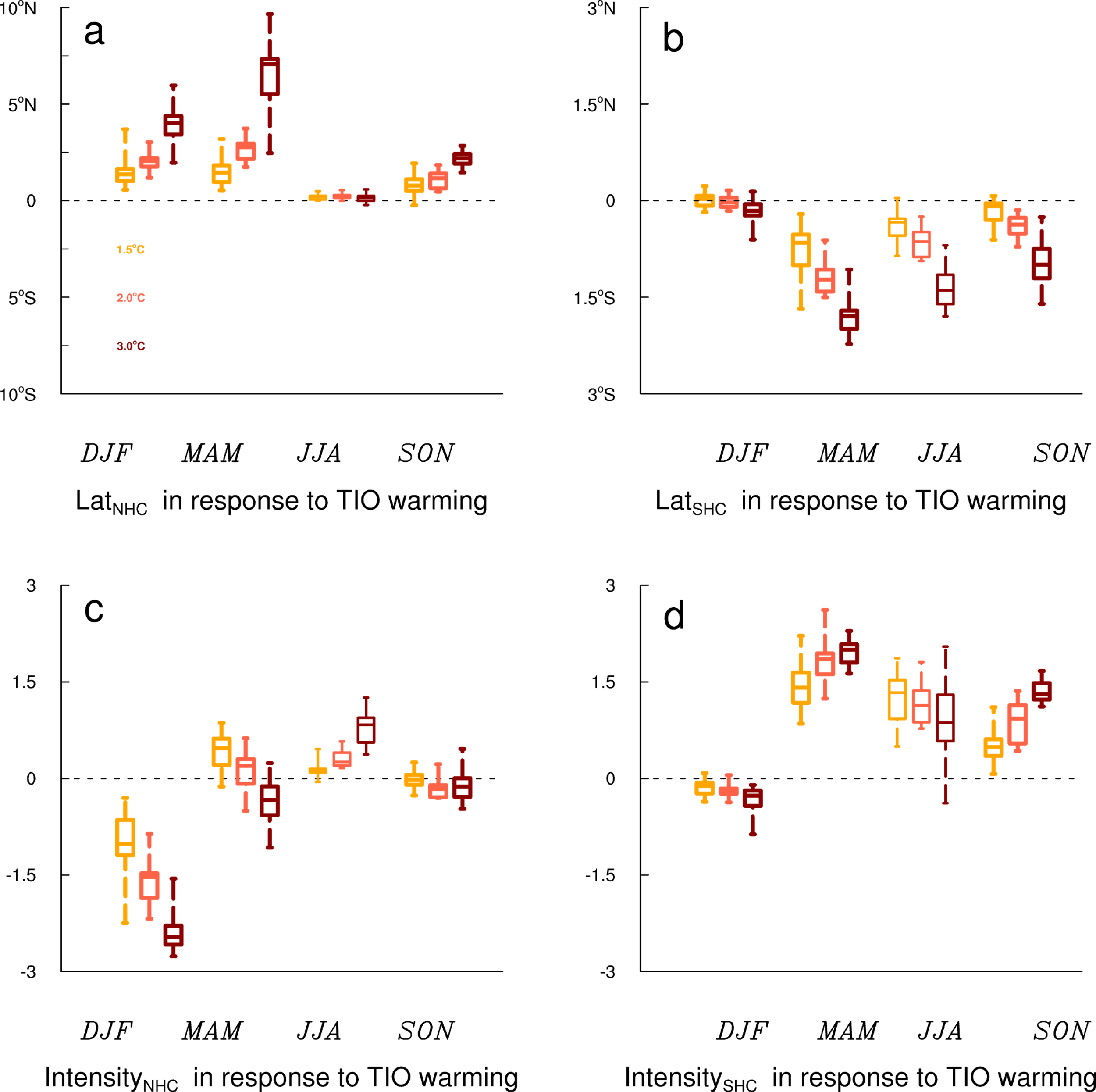


**Fig.S9** As in Fig.3, but for Lat_NHC_, Lat_SHC_ and Intensity_NHC_ and Intensity_SHC_ responses to future TIO warming patterns across the four seasons, reaching thresholds of 1.5°C, 2°C and 3°C warming.


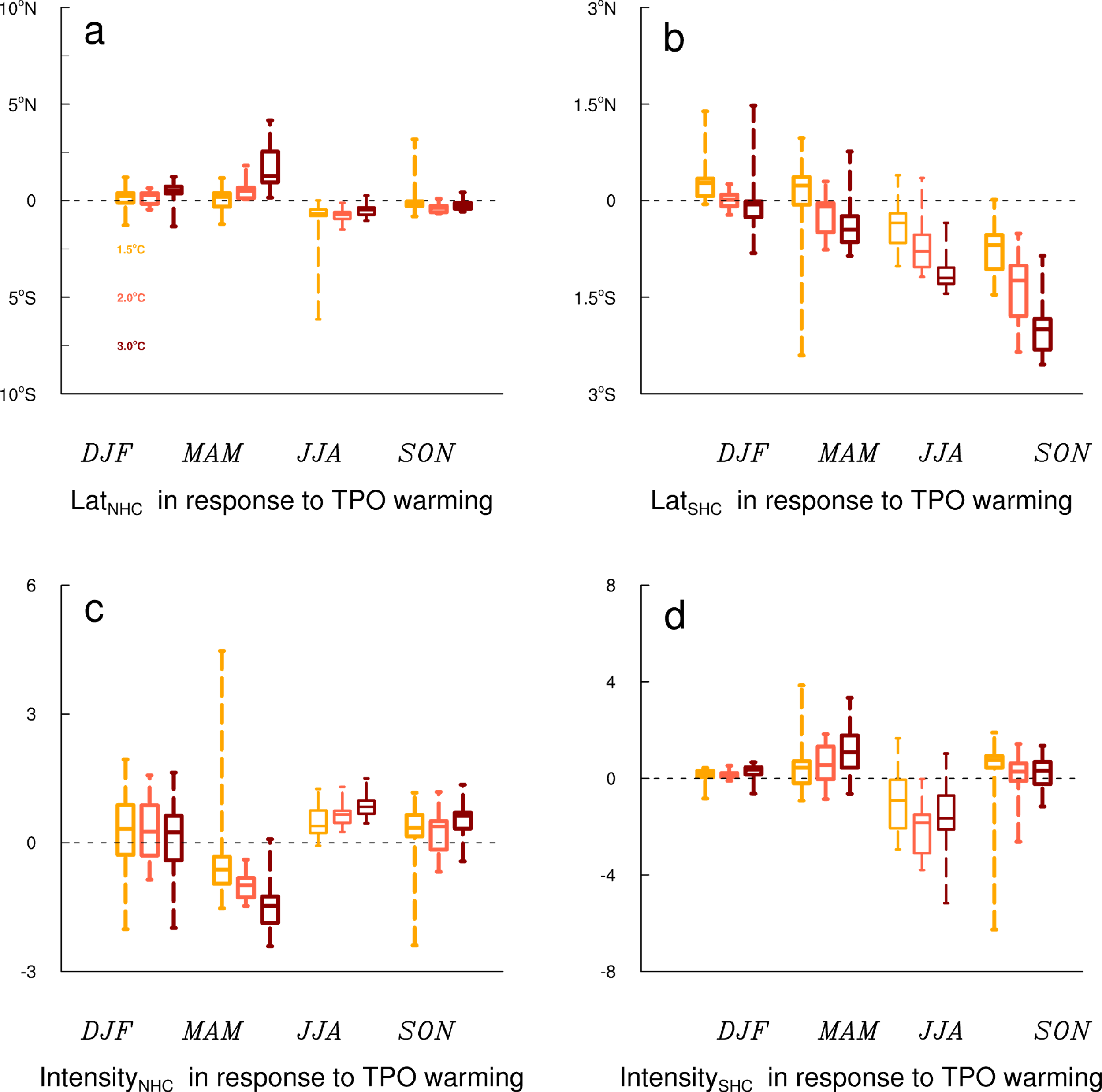


**Fig.S10** As in Fig.S9 but for Lat_NHC_, Lat_SHC_ and Intensity_NHC_ and Intensity_SHC_ responses to future TPO warming patterns across the four seasons, reaching the 1.5°C, 2°C, and 3°C warming thresholds.


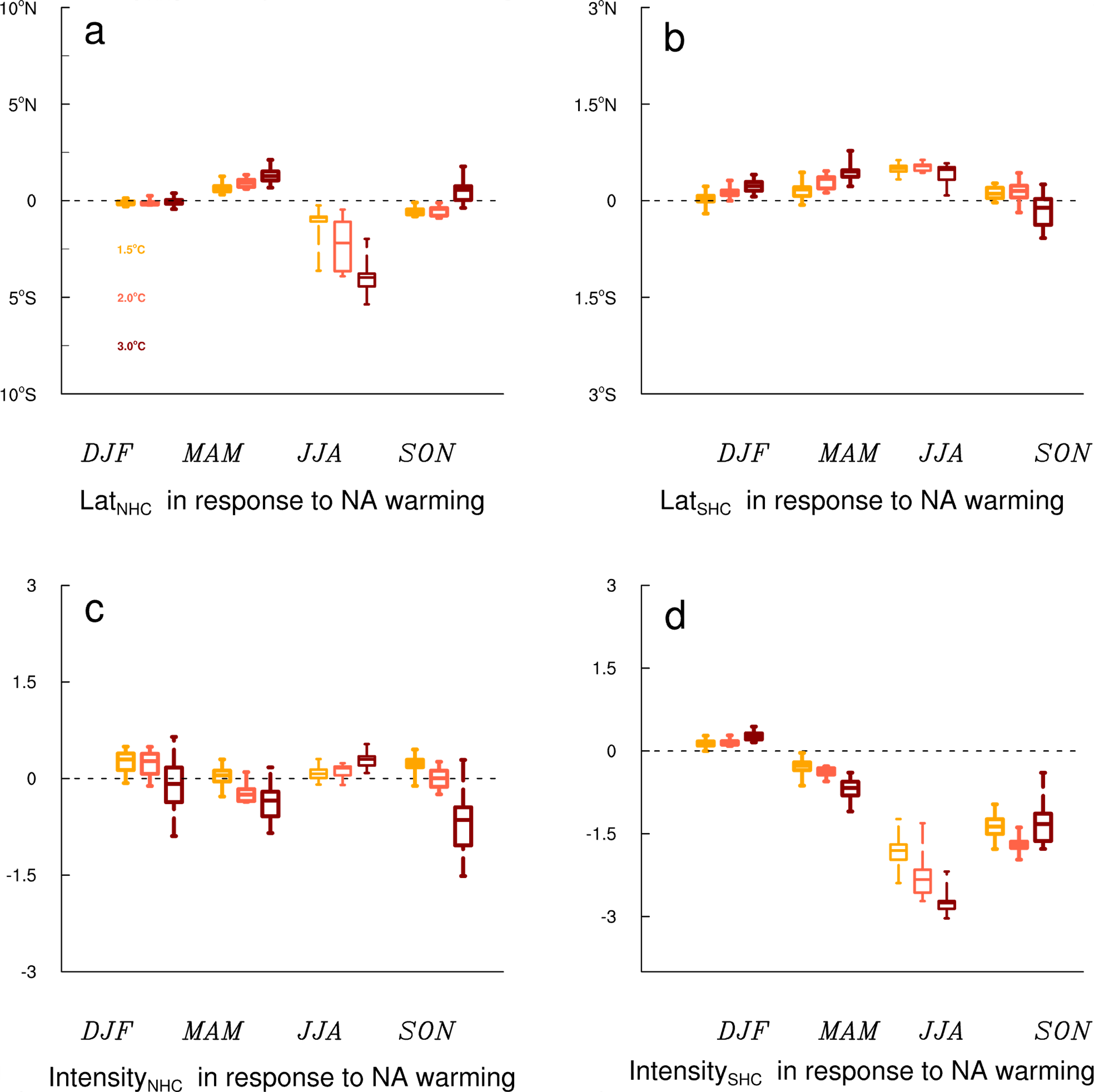


**Fig.S11** As in Fig.S9, but for Lat_NHC_, Lat_SHC_ and Intensity_NHC_ and Intensity_SHC_ responses to future NA warming patterns across the four seasons, reaching the 1.5°C, 2°C, and 3°C warming thresholds.


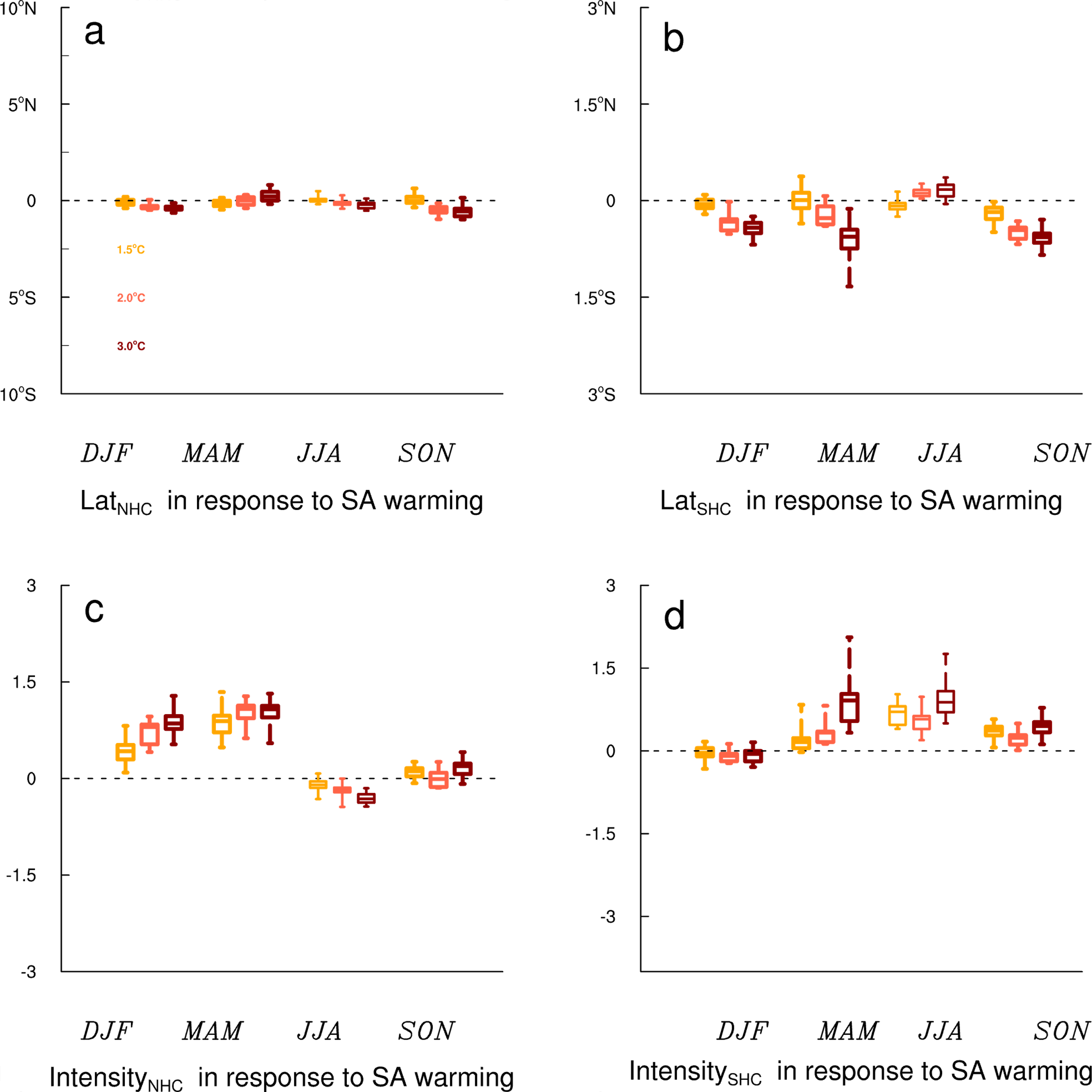


**Fig.S12** As in Fig.S9, but for Lat_NHC_, Lat_SHC_ and Intensity_NHC_ and Intensity_SHC_ responses to future SA warming patterns across the four seasons, reaching the 1.5°C, 2°C, and 3°C warming thresholds.


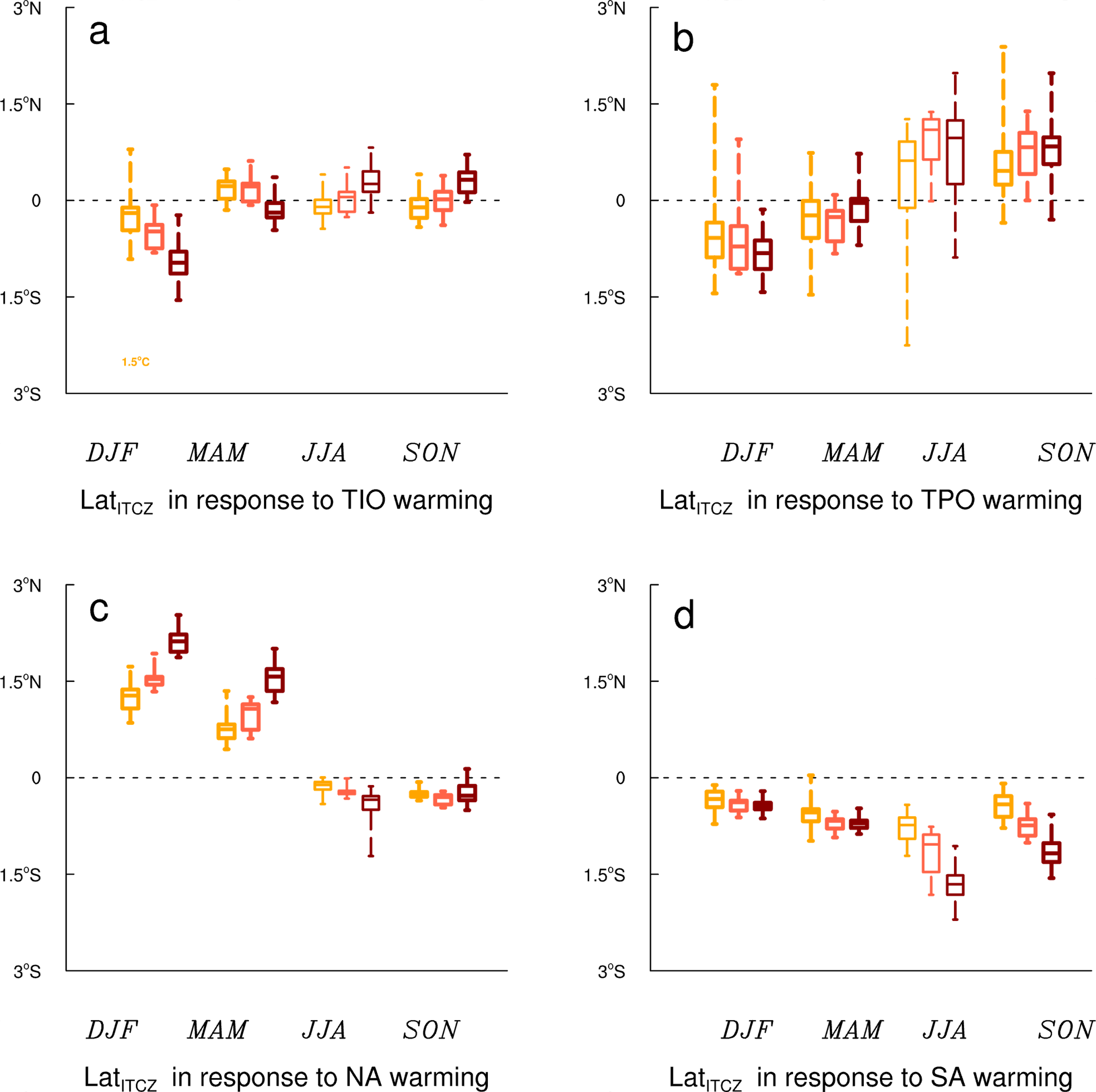


**Fig.S13** As in Fig.3, but for Lat_ITCZ_ responses to four future ocean basin (TIO, TPO, NA, SA) warming patterns across the four seasons, reaching the 1.5°C, 2°C, and 3°C warming thresholds.

**Table S1** A list of current available simulations under CMIP5 protocol used in this study to 1) to assess the relative importance of direct radiation forcing and indirect SST warming to the future projection of HC in RCP8.5 scenario. 2) obtain the SST patterns under the three temperature thresholds that are used to force the CAM4.

| Models  Experiments | | ACCESS1-0 | ACCESS1-3 | bcc-csm1-1 | bcc-csm1-1-m | BNU-ESM | CanESM2 | CCSM4 | CESM1-BGC | CESM1-CAM5 | CMCC-CMS | CNRM-CM5 | CSIRO-Mk3-6-0 | EC-EARTH | FGOALS-s2 | GFDL-CM3 | GFDL-ESM2M | GISS-E2-H | GISS-E2-H-CC | HadGEM2-AO | HadGEM2-CC | IPSL-CM5A-LR | IPSL-CM5A-MR | IPSL-CM5B-LR | MIROC5 | MIROC-ESM | MIROC-ESM-CHEM | MPI-ESM-LR | MPI-ESM-MR | MRI-CGCM3 | NorESM1-M | NorESM1-ME | FIO-ESM | GFDL-ESM2G |
| --- | --- | --- | --- | --- | --- | --- | --- | --- | --- | --- | --- | --- | --- | --- | --- | --- | --- | --- | --- | --- | --- | --- | --- | --- | --- | --- | --- | --- | --- | --- | --- | --- | --- | --- |
| piControl | | **🗸** | **🗸** | **🗸** | **🗸** | **🗸** | **🗸** | **🗸** | **🗸** | **🗸** | **🗸** | **🗸** | **🗸** | **🗸** | **🗸** | **🗸** | **🗸** | **🗸** | **🗸** | **🗸** | **🗸** | **🗸** | **🗸** | **🗸** | **🗸** | **🗸** | **🗸** | **🗸** | **🗸** | **🗸** | **🗸** | **🗸** | **🗸** | **🗸** |
| historical | | **🗸** | **🗸** | **🗸** | **🗸** | **🗸** | **🗸** | **🗸** | **🗸** | **🗸** | **🗸** | **🗸** | **🗸** | **🗸** | **🗸** | **🗸** | **🗸** | **🗸** | **🗸** | **🗸** | **🗸** | **🗸** | **🗸** | **🗸** | **🗸** | **🗸** | **🗸** | **🗸** | **🗸** | **🗸** | **🗸** | **🗸** | **🗸** | **🗸** |
| RCP4.5 | 1.5$℃$ | **🗸** | **🗸** | **🗸** | **🗸** | **🗸** | **🗸** | **🗸** | **🗸** | **🗸** | **🗸** | **🗸** | **🗸** | **🗸** | **🗸** | **🗸** | **🗸** | **🗸** | **🗸** | **🗸** | **🗸** | **🗸** | **🗸** | **🗸** | **🗸** | **🗸** | **🗸** | **🗸** | **🗸** | **🗸** | **🗸** | **🗸** |  |  |
|  | 2$℃$ | **🗸** | **🗸** |  |  | **🗸** | **🗸** |  |  | **🗸** | **🗸** | **🗸** | **🗸** | **🗸** | **🗸** | **🗸** |  |  |  | **🗸** | **🗸** | **🗸** | **🗸** | **🗸** | **🗸** | **🗸** | **🗸** | **🗸** | **🗸** | **🗸** |  | **🗸** |  |  |
| RCP8.5 | 3$℃$ | **🗸** | **🗸** | **🗸** | **🗸** | **🗸** | **🗸** | **🗸** | **🗸** | **🗸** | **🗸** | **🗸** | **🗸** |  | **🗸** | **🗸** | **🗸** | **🗸** | **🗸** | **🗸** | **🗸** | **🗸** | **🗸** | **🗸** | **🗸** | **🗸** | **🗸** | **🗸** | **🗸** | **🗸** | **🗸** | **🗸** | **🗸** | **🗸** |
| amip | |  |  | **🗸** |  |  | **🗸** | **🗸** |  |  |  | **🗸** |  |  |  |  |  |  |  | **🗸** |  | **🗸** |  | **🗸** | **🗸** |  |  | **🗸** | **🗸** | **🗸** |  |  |  |  |
| amip4$\times$CO_2_ | |  |  | **🗸** |  |  | **🗸** | **🗸** |  |  |  | **🗸** |  |  |  |  |  |  |  | **🗸** |  | **🗸** |  | **🗸** | **🗸** |  |  | **🗸** | **🗸** | **🗸** |  |  |  |  |
| amip4K | |  |  | **🗸** |  |  | **🗸** | **🗸** |  |  |  | **🗸** |  |  |  |  |  |  |  | **🗸** |  | **🗸** |  | **🗸** | **🗸** |  |  | **🗸** | **🗸** | **🗸** |  |  |  |  |
| amipFuture | |  |  | **🗸** |  |  | **🗸** | **🗸** |  |  |  | **🗸** |  |  |  |  |  |  |  | **🗸** |  | **🗸** |  | **🗸** | **🗸** |  |  | **🗸** | **🗸** | **🗸** |  |  |  |  |


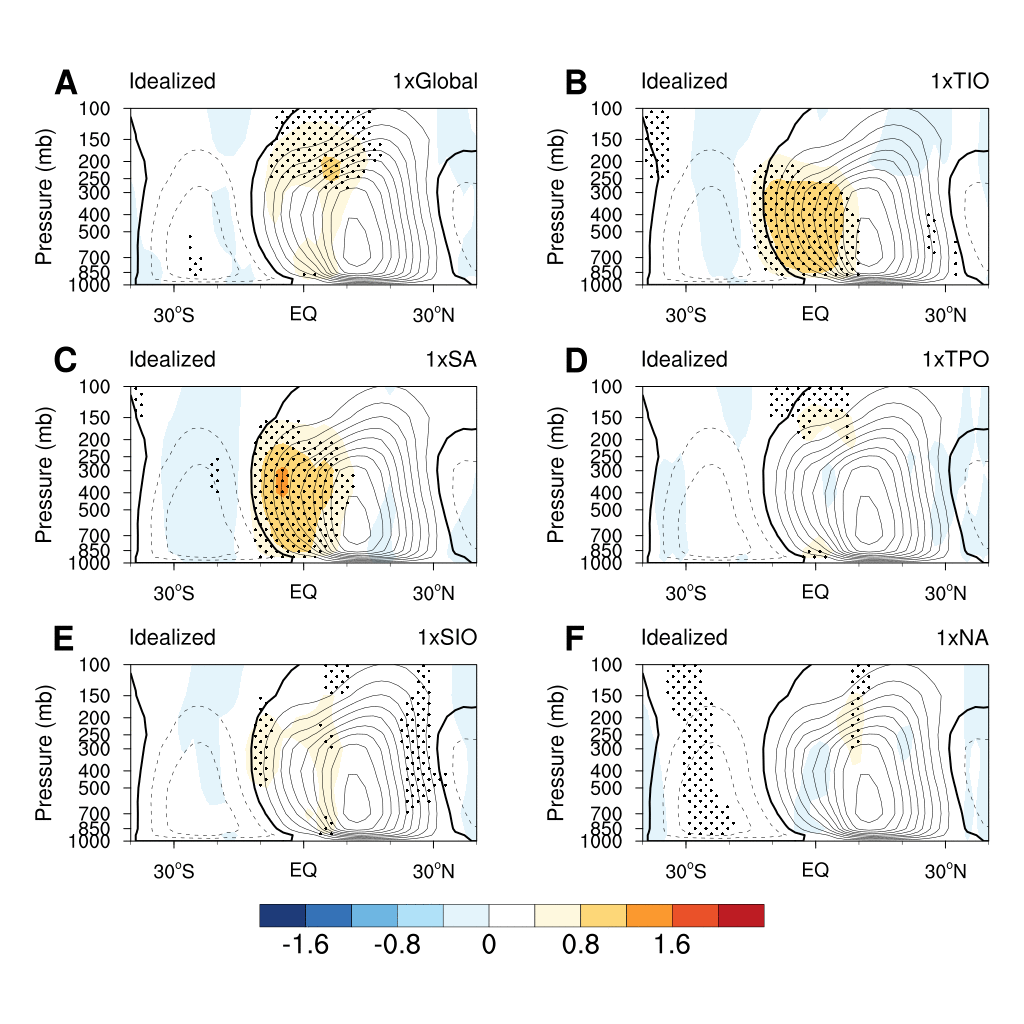


**Movie S1** A loop animation showing the responses of mass stream function (MSF) in the boreal winter to gradual amplification of global and five separate basins SST warming patterns in the idealized experiments (shading: 10^10^kg$\cdot$s^-1^) in comparison with climatology of MSF in the baseline climate (F2000, contours: 10^10^kg$\cdot$s^-1^). Dotted areas indicate responses with significance above 10%.


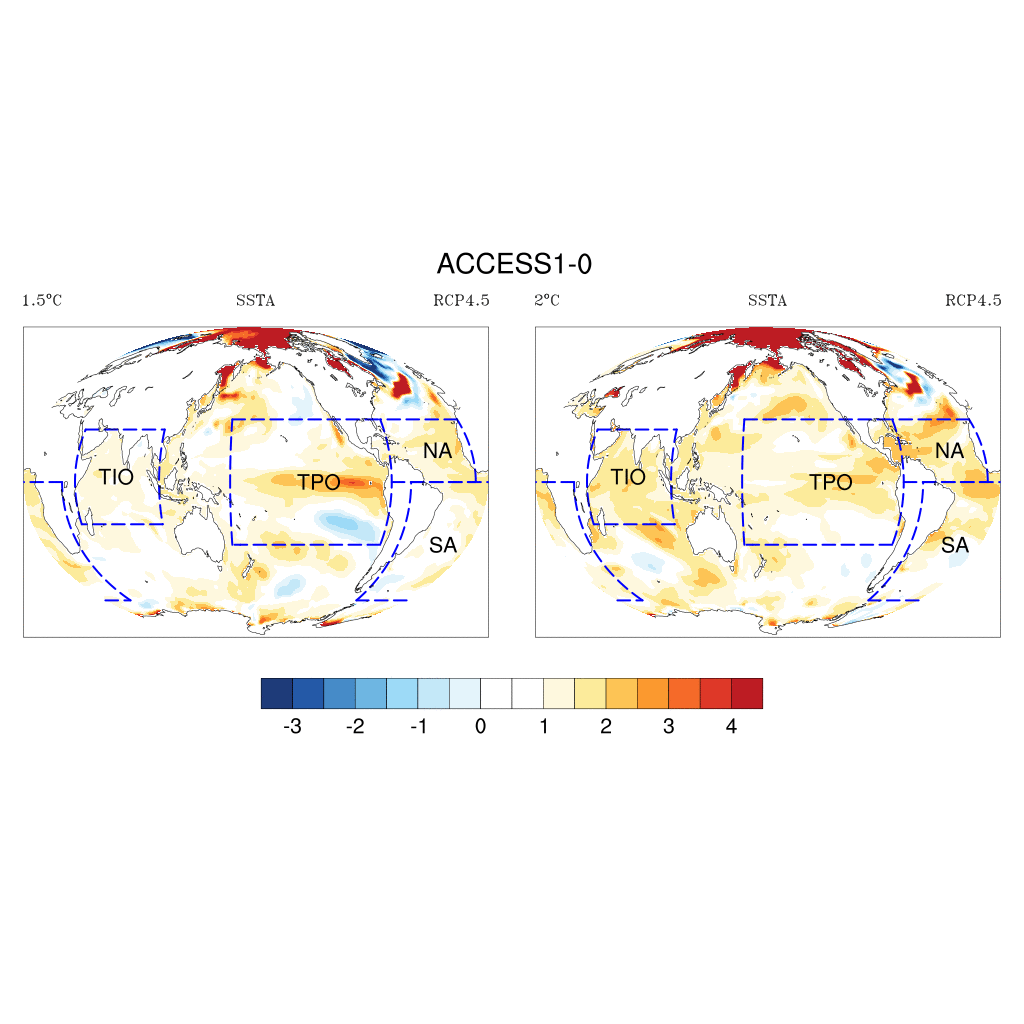


**Movie S2** The spatial patterns of future SST changes under thresholds of 1.5$℃$ and 2$℃$in the RCP4.5 scenario relative to present day (1986–2005), which are the SSTA forcings in the 1.5$℃$ and 2$℃$ target experiments for different CMIP5 models. The animation shows future projections of global SAT in the RCP4.5 scenario that reach the 1.5$℃$ and 2$℃$ thresholds above the pre-industrial level. Dashed rectangle boxes are used to distinguish different ocean basin forcings in two target experiments.


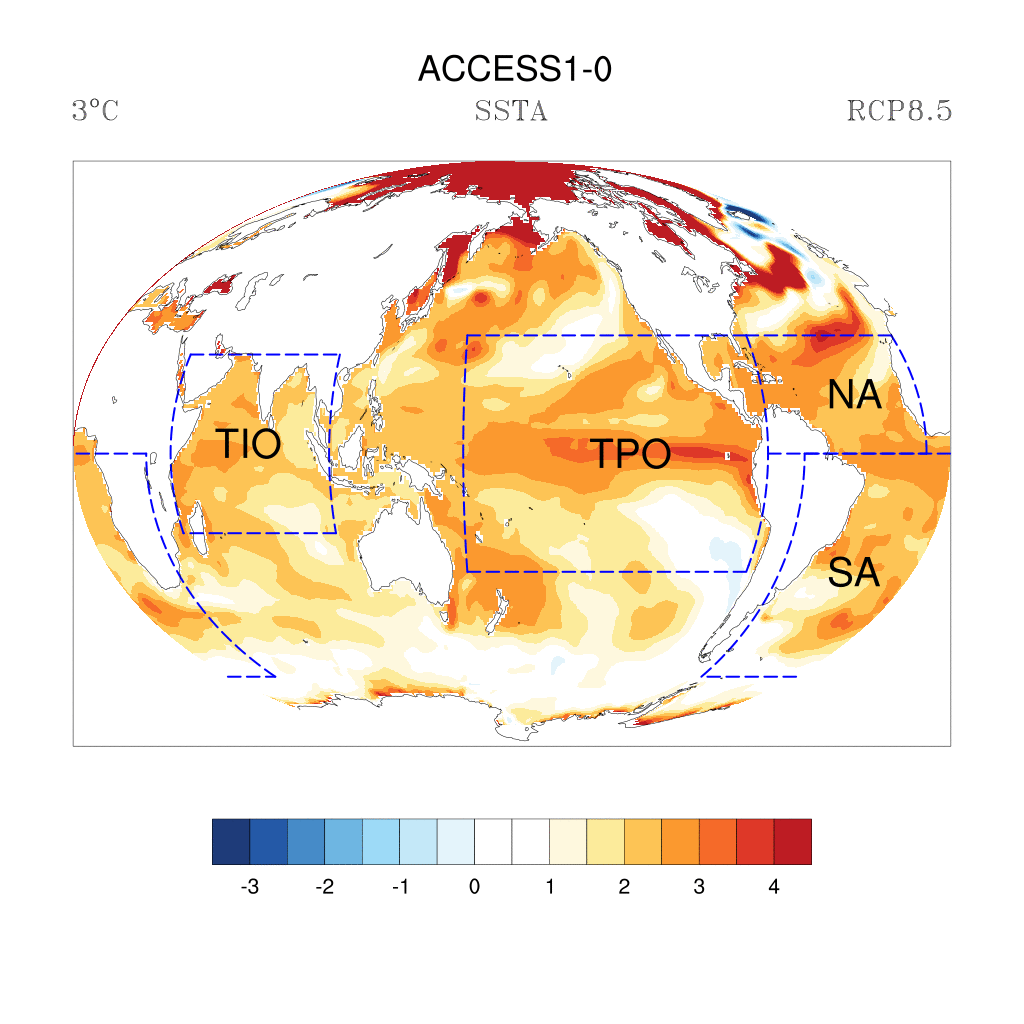


**Movie S3** As Movie S2, but showing SST warming patterns under threshold target of 3$℃$ in the RCP8.5 scenario.


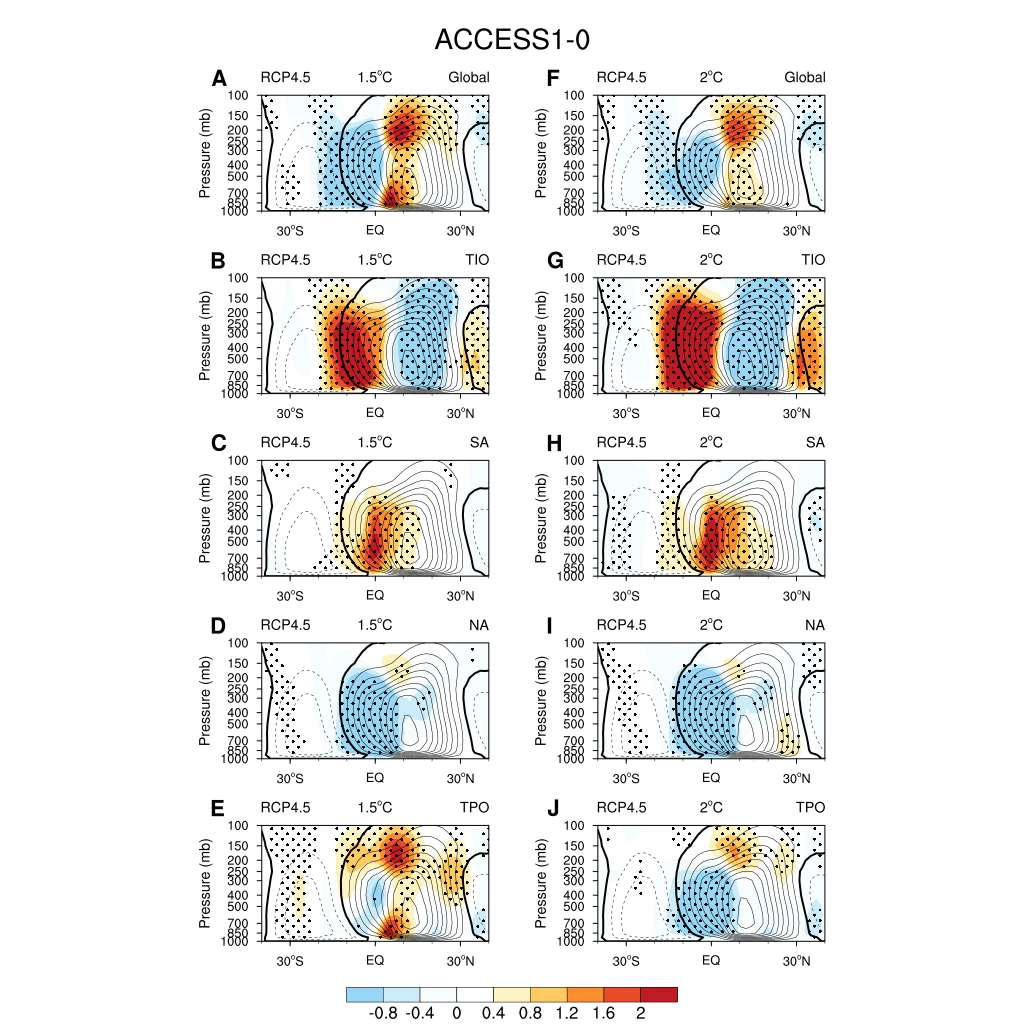


**Movie S4** A loop animation showing the responses of MSF in CMIP5 models (shading: 10^10^ kg$\cdot$s^-1^) to SSTA forcings of Global, TIO, SA, NA, and TPO under target of 1.5$℃$ and 2$℃$ in the RCP4.5 scenario. Responses with significance above 5% level are dotted, and contours indicate the climatology of MSF in present climate (10^10^ kg$\cdot$s^-1^). Red “no data” texted in some slices indicate missing responses of MSF to global and regional basin SSTA forcings under 2$℃$ in the RCP4.5 scenario due to no overshoot 2$℃$ by these model projections.


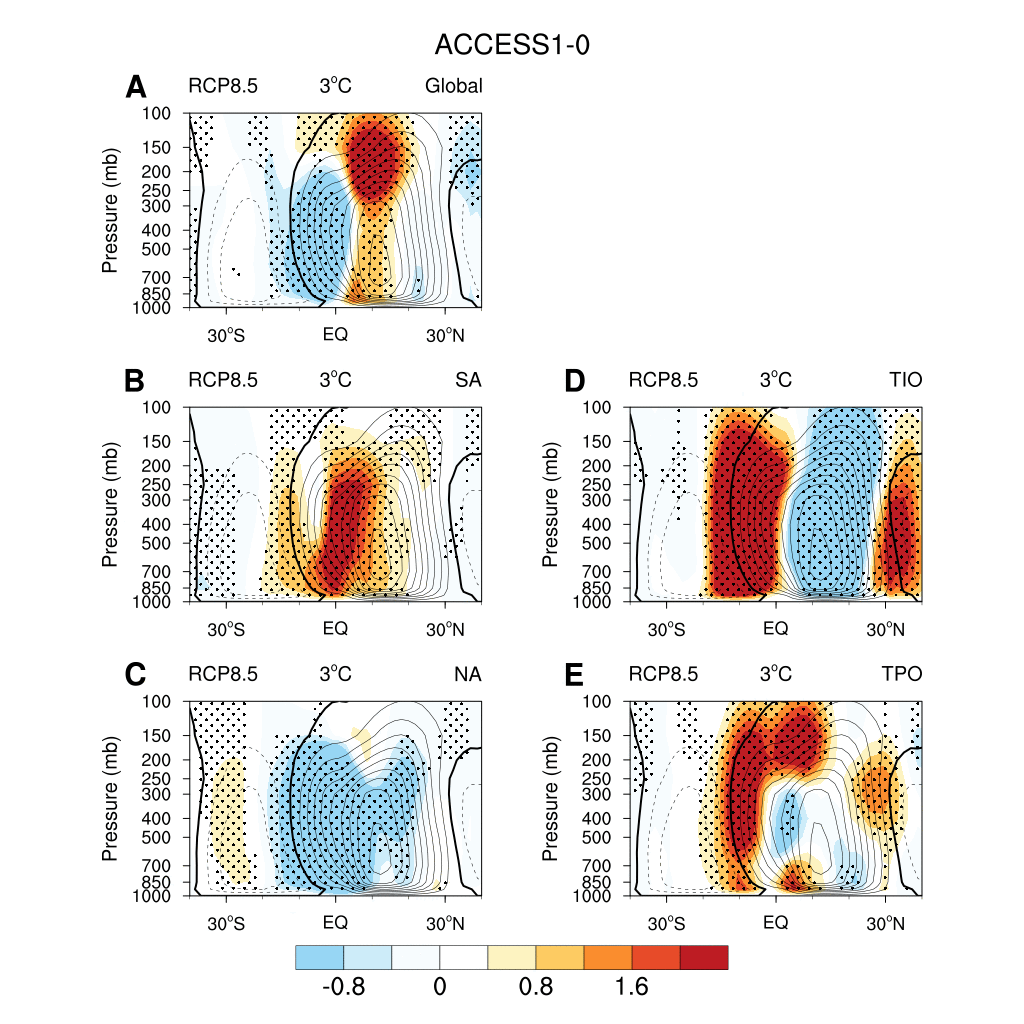


**Movie S5** As Movie S4, but showing the responses of MSF in CMIP5 models to global and four separate basin SSTA forcings under target of 3$℃$ in the RCP8.5 scenario.


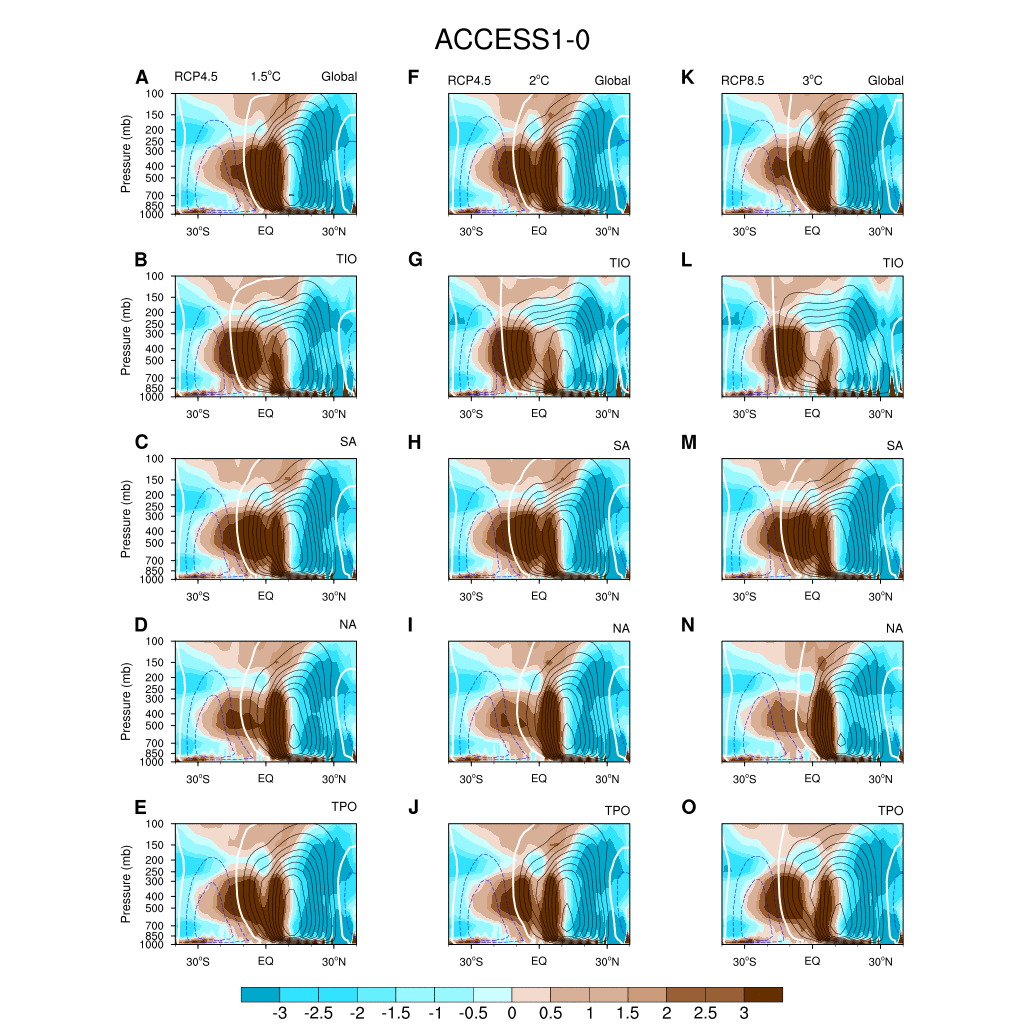


**Movie S6** As Fig.S6B, except for a spatial comparison of MSF (contours: 10^10^kg$\cdot$s^-1^) and Q (shading: K/day) under $1.5℃$, $2℃$and 3$℃$ target experiments.


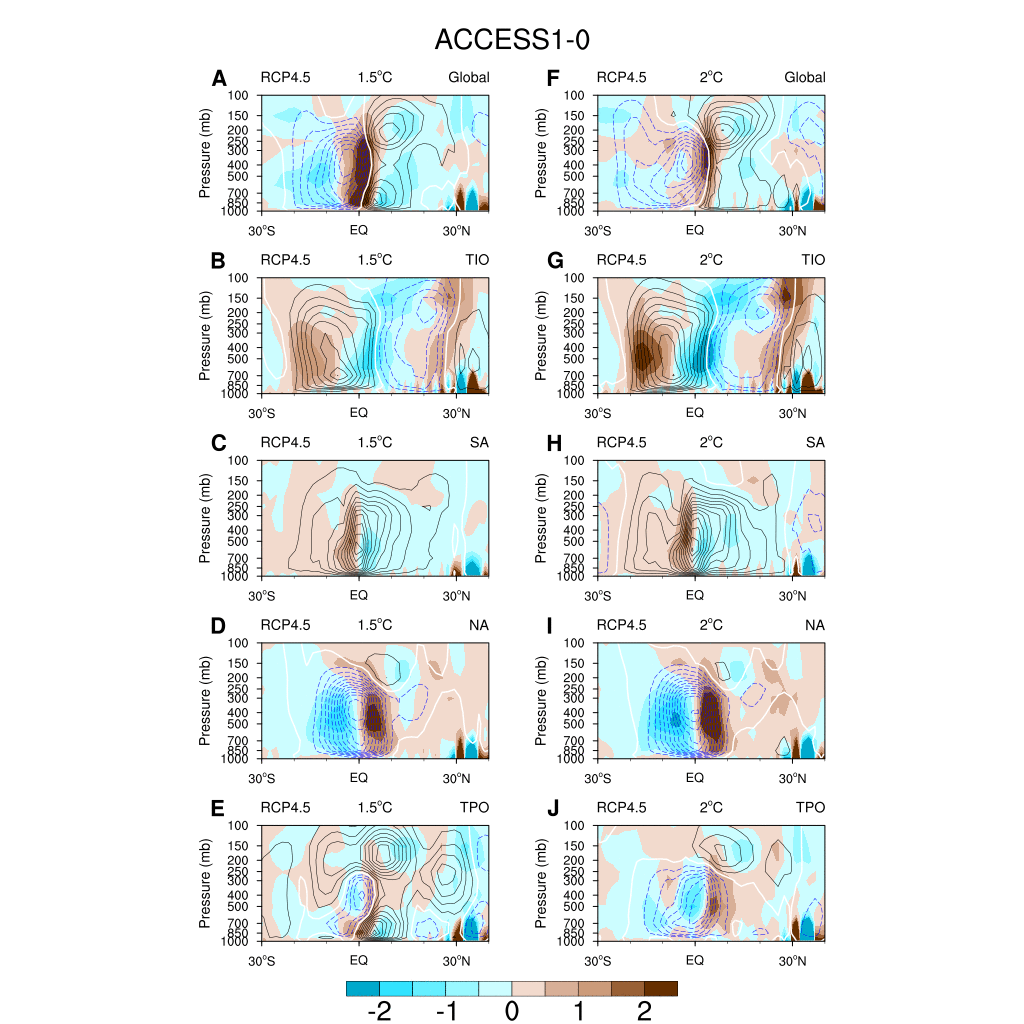


**Movie S7** As Movie S4, except for a spatial comparison of the MSF (as Movie S4, but with different contour intervals, 10^10^kg$\cdot$s^-1^) with diabatic process responses (shading: K/day) to SSTA forcings of Global, TIO, SA, NA and TPO under temperature thresholds of $1.5℃$ and 2$℃$ above the pre-industrial level.


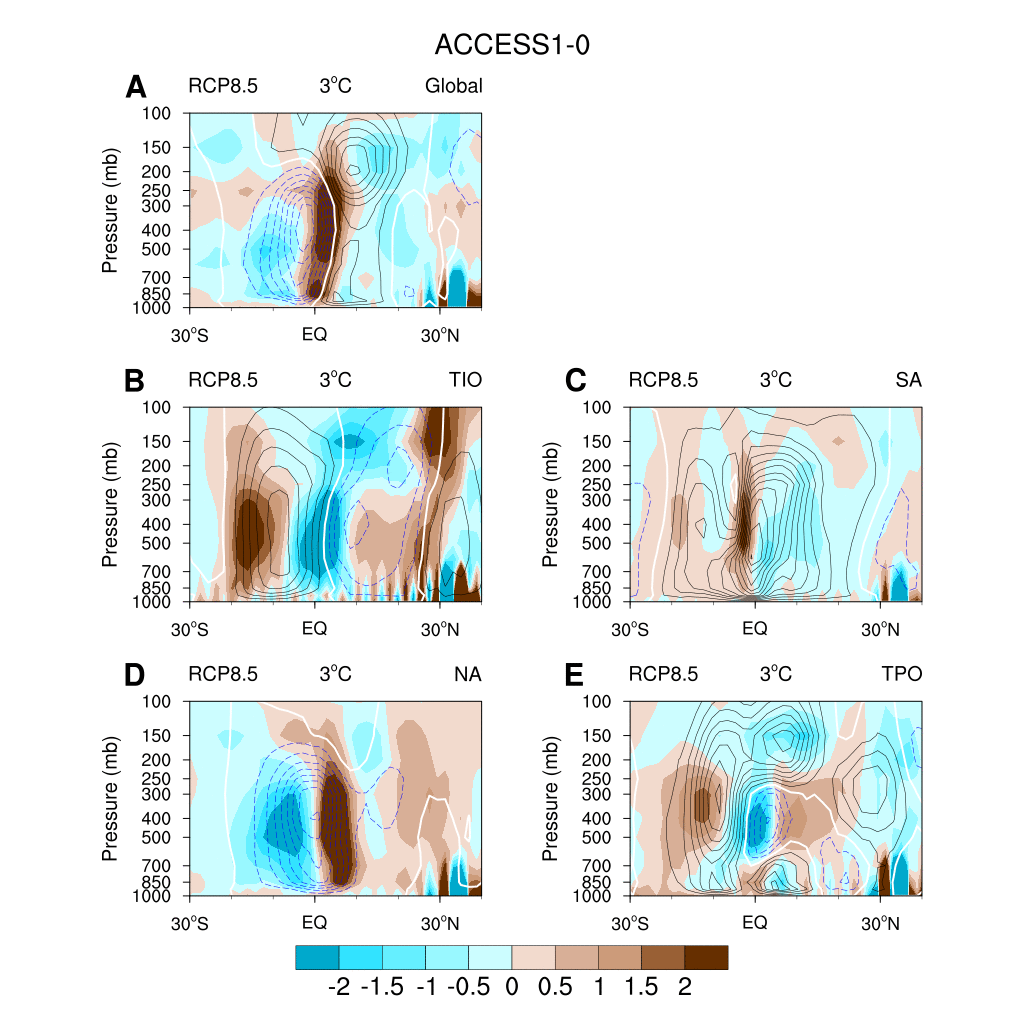


**Movie S8** As Movie S7, except for a spatial comparison of MSF with atmospheric thermodynamic structures in responses to global and four basin SST warming forcings under the threshold of 3$℃$ above the pre-industrial level.


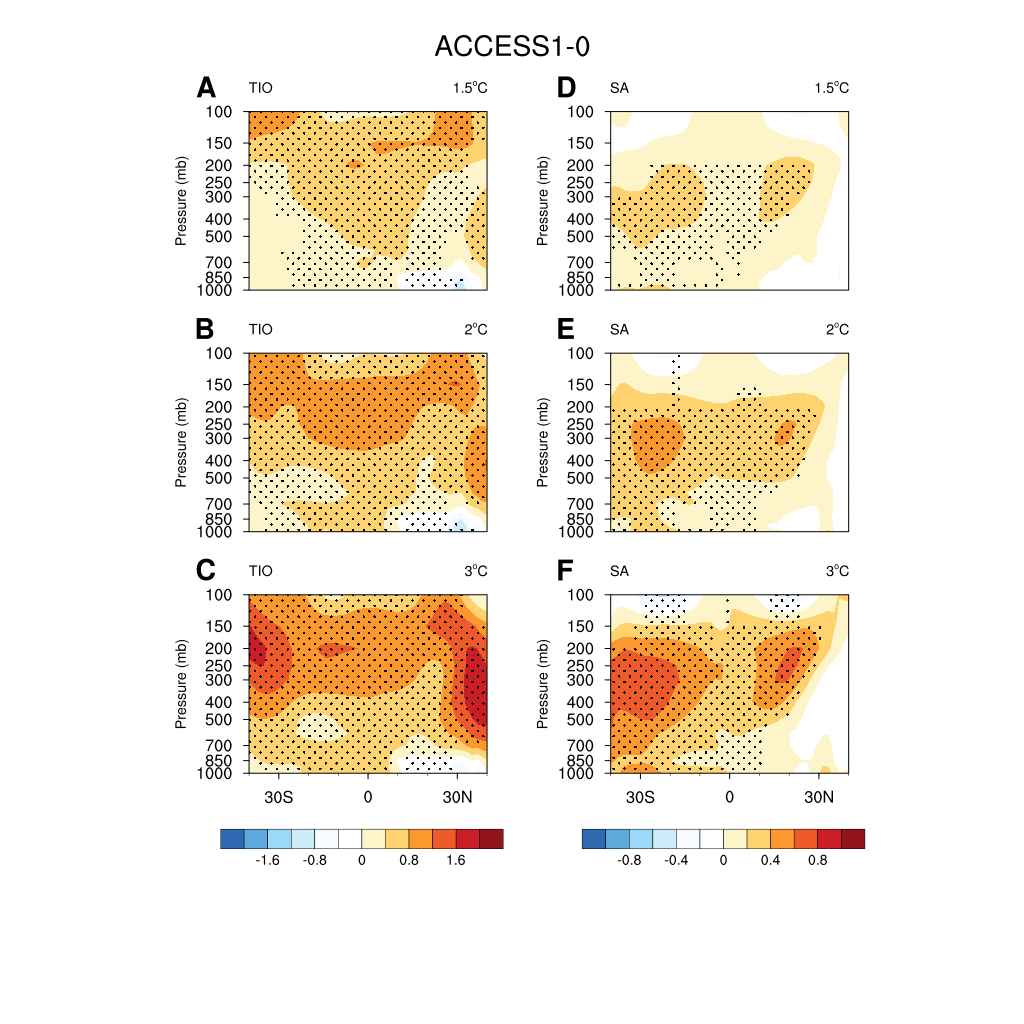


**Movie S9** A loop animation showing the responses of air temperature (shading: $℃$) to SSTA forcings of TIO and SA under target of 1.5$℃$, 2$℃$ and 3$℃$. Dotted areas indicate responses with significance above 1%.
